# Recursive seed amplification detects distinct α-synuclein strains in cerebrospinal fluid of patients with Parkinson’s disease

**DOI:** 10.1186/s40478-024-01923-8

**Published:** 2025-01-20

**Authors:** Stefan Bräuer, Iñaki Schniewind, Elisabeth Dinter, Björn H. Falkenburger

**Affiliations:** 1 Department of Neurology, University Hospital Carl Gustav Carus, Technische Universität Dresden, Fetscherstr. 74, 01307 Dresden, Germany; 2 German Center for Neurodegenerative Diseases (DZNE), Tatzberg 41, 01307 Dresden, Germany

**Keywords:** Seed amplification assay, Parkinson’s disease, Strains, RT-QuIC, Alpha-synuclein

## Abstract

Parkinson’s disease (PD) is a heterogeneous neurodegenerative disorder with a wide range of clinical phenotypes. Pathologically, it is characterized by neuronal inclusions containing misfolded, fibrillar alpha-synuclein (aSyn). Prion-like properties of aSyn contribute to the spread of aSyn pathology throughout the nervous system as the disease progresses. Utilizing these properties, seed amplification assays (SAA) enable the detection of aSyn pathology in living patients. We hypothesized that structurally distinct aSyn aggregates, or strains, may underlie the clinical heterogeneity of PD. To test this hypothesis, we recursively amplified aSyn fibrils from the cerebrospinal fluid (CSF) of 54 patients (34 people with PD and 20 controls). These fibrils were then characterized regarding SAA kinetic properties and detergent resistance. In addition, cultured cells were transfected with SAA products, and the extent of seeded aSyn pathology was quantified by staining for phosphorylated aSyn followed by automated high-throughput microscopy and image analysis. We found that fibrils, amplified from CSF by recursive SAA, exhibit two types of distinct biophysical properties and have different seeding capacities in cells. These properties are associated with clinical parameters and may therefore help explain the clinical heterogeneity in PD. Measuring aSyn strains may be relevant for prognosis and for therapies targeting aSyn pathology.

**Supplementary Information:**

The online version contains supplementary material available at 10.1186/s40478-024-01923-8.

## Introduction

Lewy bodies and neurites containing aggregated alpha-synuclein (aSyn) represent the neuropathological hallmark of Parkinson’s disease (PD) [44]. Unfortunately, clinical trials of antibodies targeting aSyn pathology have failed to meet their primary endpoints [25, 31]. The recent observation that only a subset of patients may benefit from prasinezumab [32] suggests that heterogeneity of patients with PD could contribute to these negative trials. Heterogeneity in PD has been recognized for a long time, discriminating between early-onset and late-onset, tremor-dominant (TD) and postural instability and gait difficulty (PIGD) subtypes [23]. More refined classification systems were subsequently based on the presence of orthostatic hypotension, cognitive impairment, rapid eye movement sleep behavior disorder (RBD), depression, anxiety and the severity of motor symptoms as assessed by the scores in the Unified Parkinson’s Disease Rating Scale, discriminating a “diffuse-malignant” subtype with pronounced non-motor symptoms and rapid progression from “mainly motor / slow progression” and intermediate [13]. The causes of this clinical heterogeneity have remained largely unclear, even though differences in genetics [11] and the origin of aSyn pathology have been suggested [5, 20].

In prion disorders, the concept of strains was first introduced to describe the phenomenon that, in scrapie, aggregates of the same protein can cause different clinical and neuropathological phenotypes [3, 14]. Distinct conformations of the protein monomer in the fibril are considered to be the structural correlate of strains [3]. These conformations are amplified when prions act as seeds for the misfolding of additional monomers. There is already evidence from previous reports that distinct aSyn strains might be involved in different disorders, namely PD and multiple system atrophy (MSA) [41].

Seed amplification assays (SAA) have been established to detect aggregated proteins in biomaterials. Initially developed for the prion protein [28], these assays have been adapted to prion-like proteins; they are especially well established for the detection of aggregated aSyn in PD and dementia with Lewy bodies [12, 16, 40]. The possibility to detect aSyn pathology in living patients via SAA has enabled a biological definition of neuronal aSyn diseases [19, 42].

We hypothesized that SAA can be used to test whether aSyn strains contribute to the clinical heterogeneity of PD. To this end, we collected cerebrospinal fluid (CSF) from 54 patients (34 people with PD and 20 controls) and recursively amplified aSyn seeds via SAA. The amplified aSyn fibrils differed in biophysical characteristics and biological properties, such as the capacity to seed aSyn pathology in cells and the clinical phenotype of CSF donors. Using recursive seed amplification, we were thus able to detect two different strains in what is considered a single disorder, PD. Our findings support the hypothesis that strains of aSyn pathology contribute to the clinical heterogeneity in PD.

## Materials and methods

### Study population

Cerebrospinal fluid (CSF) was collected from consenting patients with Parkinson’s disease (PD) at the Department of Neurology, University Clinic Dresden, who underwent lumbar puncture as part of routine diagnostics between 2021 and 2024. Patients were diagnosed by a movement disorder specialist according to the MDS criteria [38]. Lumbar punctures were performed according to standard protocols. CSF samples were centrifuged and frozen at -80 °C within 90 min of collection. The study was approved by the local ethics committee (BO-EK-444092021).

MDS-Unified Parkinson’s Disease Rating Scale (UPDRS) data were extracted from patient records. Whenever possible, MDS-UPDRS part III was assessed in motor OFF states (26 of 34 PD patients). Motor phenotype classification (postural instability and gait difficulty (PIGD) vs. tremor dominant (TD)) followed previously established methods [45]. The Montreal Cognitive Assessment (MoCA) was administered as part of routine clinical evaluations. Olfactory function was assessed using Sniffin’ Sticks (Burghart Messtechnik) [21]. RBD was assessed during patient interview [47], using either the RBD screening questionnaire (RBDSQ) [46] or the RBD Single-Question Screen (RBD1Q) [37]. Depression was diagnosed by a specialist according to clinical guidelines. Information regarding constipation and sleep disturbances was gathered from patients’ medical histories. Patient demographics are summarized in Supplementary Table 1.

### Purification of human α-synuclein

The aSyn purification was performed as described for the “fast batch” aSyn [8]. Briefly, BL21 (DE3) E. coli bacteria (Thermo Fischer Scientific) were transformed with the vector plasmid containing WT human aSyn. The aSyn ORF was cloned from pT7-7 aSyn WT (gift from Hilal Lashuel, Addgene plasmid #36046) into pET-28a(+) (Merck) using the restriction sites HindIII and NdeI. Expression was induced using autoinduction medium, and cells were harvested after 16 h. The cell pellet was lysed via osmotic shock [400 g/l sucrose (Carl Roth), 30 mM TRIS (Carl-Roth) pH 7.2, 2 mM EDTA (Thermo Fischer Scientific)]. The solution was centrifuged and the pellet resolved in water. After centrifugation, the supernatant’s pH was reduced to 3.5 using HCl. The solution was centrifuged again and the supernatant’s pH was increased to 7.5 using NaOH. Next, we performed immobilized metal ion affinity chromatography using an NGC chromatography system (BioRad) and HisTrap FF-column (Cytivia). The selected fraction was loaded on a HiTrap Q-HP anion exchange-column (Cytivia), the selected fractions pooled and subsequently dialyzed against water using a 3.5 kDa MWCO dialysis membrane (Thermo Fisher Scientific) overnight at 4 °C. The protein concentration was measured using a NanoDrop spectrophotometer (Cat# ND-LITE-PR, Thermo Scientific). Using this protocol, the average yield from 1 L of bacterial culture was ~ 65 mg aSyn. The protein was aliquoted in 0.7 mg portions and stored at − 80 °C until further use.

### α-synuclein seed amplification assay

The aSyn SAA was performed as previously described [8]. In brief, measurements were performed in a black 96-well plate (Thermo Fischer Scientific). Each well contained six 0.8 mm silica beads (OPS Diagnostics). Per well, 15 µl of CSF was added to 85 µl of reaction buffer, which contained 40 mM phosphate buffer (Carl Roth) pH 8.0, 0.0015% sodium dodecyl sulfate (SDS, Carl Roth), 10 µM Thioflavin T (Carl Roth), 0.1 mg/ml recombinant aSyn, 170 mM NaCl (Carl Roth). The plate was incubated in a BMG FLUOstar Omega plate reader at 42 °C with alternating cycles of 1 min double orbital shaking (400 rpm) and 1 min rest. The fluorescence measurements were performed every 45 min.

Each sample was measured in four technical replicates on the same plate. For the initial amplification from the CSF, each plate included at least two negative and two positive controls, each in four replicates. Relative fluorescence units (RFU) for every time point were expressed as a percentage of the maximum intensity reached on that plate. A replicate was considered positive if fluorescence crossed a defined threshold within a 40-hour time window. The fluorescence threshold was defined as the average intensity of previously measured negative controls during the first 10 h of recording, plus 40 standard deviations. A sample was considered positive if at least two replicates were positive and was considered negative if no replicate was positive. A sample was run again if one replicate was positive. In this study no sample had to be run twice. In positive samples, the following kinetic parameters were determined for each positive replicate: Area under the curve (AUC), peak of the fluorescence response (Imax), lag phase (LAG), i.e., the time to reach the threshold. For each sample, these parameters were summarized by calculating the mean of all positive replicates. The parameter TT2 (“Time to 2 positive replicates”) was defined as the second shortest LAG as previously described [8].

For the detection of the strain type of a CSF sample, the aggregates from every positive replicate were harvested by repeated up-and-down-pipetting and pooled for each sample after 70 h of amplification. We paid close attention to avoid cross contamination. Furthermore, all steps of the rSAA were performed in a cell culture hood to avoid contaminations. For the second amplification round (Amp2) the aggregates were diluted 1:1,000 in PBS. The SAA was run under the same conditions as described above except that we used 15 µl of the diluted fibrils instead of CSF. Again, each sample was measured in four technical replicates on the same plate. Amp2 products were harvested after 40 h of amplification. After fibril purification (described in a separate section) the resulting solutions were then used for the third round of amplification (Amp3). The SAA was again performed under the same conditions as Amp2. In the manuscript, this procedure (CSF-> Amp1-> Amp2-> Amp3) is referred to as rSAA. We ran the same control fibrils for both kinetic types in every rSAA performed. Those fibrils were Amp1 products for which we already measured the kinetic type. To evaluate the kinetic type, we averaged the RFU values of the four replicates of each respective sample. We then calculated the AUC using the MARS software (version 4.0 R2, BMG) in the 8-hour interval beginning after the first RFU value on the plate exceeded 10,000 RFU (Supplementary Fig. 1). A fibril with an AUC greater than 40% of the maximum average AUC in the 8-hour interval was defined as fast kinetic, fibrils with a lower AUC were defined as slow kinetic. We refer to the result of a single rSAA as the kinetic type of a fibril. The kinetic type of a CSF sample is defined by the result of multiple rSAA. After measuring the first 14 CSF samples in the rSAA, we decided to define a CSF sample as “fast kinetic” when at least 40% of independent rSAA resulted in fast aggregation kinetics in Amp3. We initially thought about defining a sample as fast kinetic when at least one rSAA resulted in this kinetic type (this case did not occur in the measurements of our samples). However, since we could not exclude that fast kinetic fibrils might contaminate a slow kinetic sample during the assay procedure, we decided that at least 2 out of 5 rSAA (40%) need to be fast kinetic. This cut-off needs to be validated in future studies.

Taken together, the procedure described resulted in a final dilution of the CSF/initial aggregates of at least 1:30,000,000,000 in Amp3.

### α-synuclein batch quality control for strain detection

The reproducible amplification for strain detection from the CSF (Amp1) required aSyn monomer of particularly high quality. We therefore implemented a stringent protocol for quality control. Quality was assessed by measuring at least 6 positive and 6 negative CSF controls in four replicates on the same plate. For an aSyn monomer to be considered “high quality”, at least three of the four replicates of all positive controls had to reach the threshold in 30 instead of 40 h. Additionally, in all of the at least 24 replicates of the negative controls, only one replicate was allowed to show aggregation during an 80-hour period (Supplementary Fig. 2).

### Modified conformational stability assay

We performed a modified conformational stability assay based on the method described by Lau et al. [26]. Equal volumes of GdnHCl stocks were added to 20 µl of Amp3-fibrils (0.25 mg/ml) resulting in GdnHCl concentrations of 0.5 M, 1 M, 1.5 M, 2 M, 2.5 M, 3 M, 3.5 M, and 4 M. For the 0 M GdnHCl sample, 20 µl of pure water was added. We incubated the samples under continuous shaking at 800 rpm for 2 h at room temperature (RT). Subsequently, the GdnHCl concentration was adjusted to 0.4 M resulting in same total volumes and ThT was added to a final concentration of 50 µM. Samples were loaded onto a black 96-well plate in technical duplicates or more. Fluorescence was measured using a BMG FLUOstar Omega plate reader every 5 min for 6 h at 25 °C with alternating cycles of 1 min of double orbital shaking at 700 rpm and 1 min of rest. The RFU values of the replicates were averaged over a three-hour interval for each condition. GdnHCl_50_ values, representing the GdnHCl concentration at which 50% of the fibrils are denatured, were calculated using nonlinear regression with the dose-response function (variable slope) in GraphPad Prism (v.10.2.2). For this, values were normalized to the 0 M GdnHCl condition (= 100%) after subtraction of the ThT control value. For statistical analysis, GdnHCl_50_ values were calculated for each replicate and compared using a two-tailed, unpaired *t* test.

### HEK293T cell culture

HEK293T cells (Cat# CRL-3216, ATCC, RRID: CVCL_0063) were cultured in Dulbecco’s Modified Eagle Medium (DMEM, high glucose, GlutaMAX™, Cat# 31966021, Gibco) supplemented with 10% fetal bovine serum (Cat# AC-SM-0143, anprotec) and 1% penicillin/streptomycin (Cat# 15140122, Gibco) in a humidified incubator at 37 °C and 5% CO_2_.

To generate stable clones overexpressing aSyn, we introduced an aSyn-T2A-GFP construct [49] into HEK293T cells through lentiviral transduction. This construct enables the coexpression of wild-type human aSyn and green fluorescent protein (GFP) as the T2A sequence facilitates the cleavage of the two proteins during translation [50]. Following transduction, single cells expressing GFP were isolated using a BD FACS Aria III (BD Biosciences) and expanded. Clones used for further experiments were selected based on comparable proliferation rates and fluorescence homogeneity.

### Purification of fibrils amplified by seed amplification assay

To isolate the aSyn fibrils amplified by SAA from patient samples, the fibril solutions were transferred into Amicon™ Ultra-0.5 Centrifugal Filter Units with a molecular weight cutoff of 100 kDa (Cat# UFC5100, Merck Millipore) and centrifuged at 14,000 G for 10 min, followed by two washes with phosphate buffered saline (PBS). The filters were then inverted into fresh tubes and centrifuged at 2,000 G for 1 min. After a portion of the fibril solution was converted to monomeric protein form in 4 M guanidinium chloride (GdnHCl, Cat# G3272, Sigma-Aldrich) for 2 h at RT and 800 rpm, the protein concentration was determined using a NanoDrop spectrophotometer (Cat# ND-LITE-PR, Thermo Scientific) by measuring absorbance at 280 nm with a molar extinction coefficient of 5,960 M^− 1^ cm^− 1^. Finally, the fibril solutions were adjusted to equal concentrations using PBS and stored at RT until further use.

### Generation of pre-formed fibrils

Pre-formed fibrils (PFF) were generated by shaking α-synuclein (aSyn) monomer (Cat# S-1001-2, rPeptide) at a concentration of 5 mg/ml in a buffer containing 50 mM Tris-HCl and 150 mM KCl, pH 7.5, at 1000 rpm and 37 °C for 1 week [43]. The resulting turbid solution was centrifuged at 21,130 G for 10 min. The supernatant was discarded, and the fibrils were resuspended in the same buffer. Protein concentration was measured using a NanoDrop spectrophotometer (Cat# ND-LITE-PR, Thermo Scientific), and PFF were diluted to a final concentration of 5 mg/ml. PFF were aliquoted and stored at -80 °C until further use.

### Fibril sonication

Fibril solutions were sonicated in 1.5 ml sonication tubes (Cat# 53071, Active Motif) for 20 min at 70% amplitude using a Qsonica Q700 sonicator with a cup horn (Cat# 431C2, Qsonica) and a connected chiller (Cat# 4905 − 110, Qsonica) set at 20 °C. Sufficient and comparable fragmentation, i.e., an average radius less than 50–60 nm, was confirmed for each sample by dynamic light scattering (DLS) using a Zetasizer Nano ZS (Malvern Panalytical). Insufficiently fragmented samples were excluded.

### Fibril transfection

HEK293T-aSyn-T2A-GFP clones were seeded at a density of 2,500–3,500 cells per well in black 96-well plates (Cat# 6055302, PerkinElmer). After 24 h, cells were transfected with freshly sonicated patient fibrils at a final concentration of 500 ng/mL using Metafectene (Cat# T020, Biontex) at a ratio of 1 µg of fibrils to 3 µL of Metafectene. Cells were fixed in 4% paraformaldehyde 48 h after fibril transfection. Cell experiments were conducted in three stable cell clones with at least three biological replicates per clone. Each biological replicate included three technical replicates.

### Immunofluorescence staining

After fixation, the cells were washed in Tris-buffered saline (TBS), permeabilized with 0.2% Triton X-100 in TBS (v/v) for 10 min, and then incubated in blocking buffer (TBS supplemented with 5% donkey serum) at RT for 1 h. The cells were then exposed to primary rabbit anti-alpha-synuclein (phospho S129) monoclonal antibody (1:1000, Cat# ab51253, abcam, RRID: AB_869973) in blocking buffer for two hours at RT. After TBS washes, the cells were incubated with Alexa Fluor 555 donkey anti-rabbit secondary antibody (1:1000, Cat# A-31572, Invitrogen, RRID: AB_162543) in the blocking buffer for another two hours at RT. Finally, the nuclei were stained with Hoechst (Cat# H3570, Invitrogen).

### Imaging and image analysis

Cells were imaged using an automated spinning disk confocal microscope (CV7000, Yokogawa). For each well, 8 fields were imaged using a 20x objective, resulting in the acquisition of a total number of more than 280 images and 150,000 cells per patient sample (Supplementary Table 2). Subsequent image analysis was performed using CellProfiler [48].

### Statistical analysis

Data analysis was conducted using GraphPad Prism Software (v10.2.2) and R Statistical Software (v4.2.1). A *P* value ≤ 0.05 was considered statistically significant. Details on statistical tests are provided in the respective figure legends.

## Results

### Recursive seed amplification of aSyn fibrils reveals two distinct types of aggregation kinetics

In SAA, a small sample of biomaterial, such as 15 µl of CSF or biopsy material, is added to a solution of aSyn monomer. This solution is then incubated for 30–70 h with repetitive agitation. Buffer conditions are chosen to ensure that aSyn only aggregates if the biomaterial contains aSyn seeds. During SAA, aSyn aggregation is reported by the fluorescent dye thioflavin T (ThT), which interacts with beta-sheet structures.

**Fig. 1:**
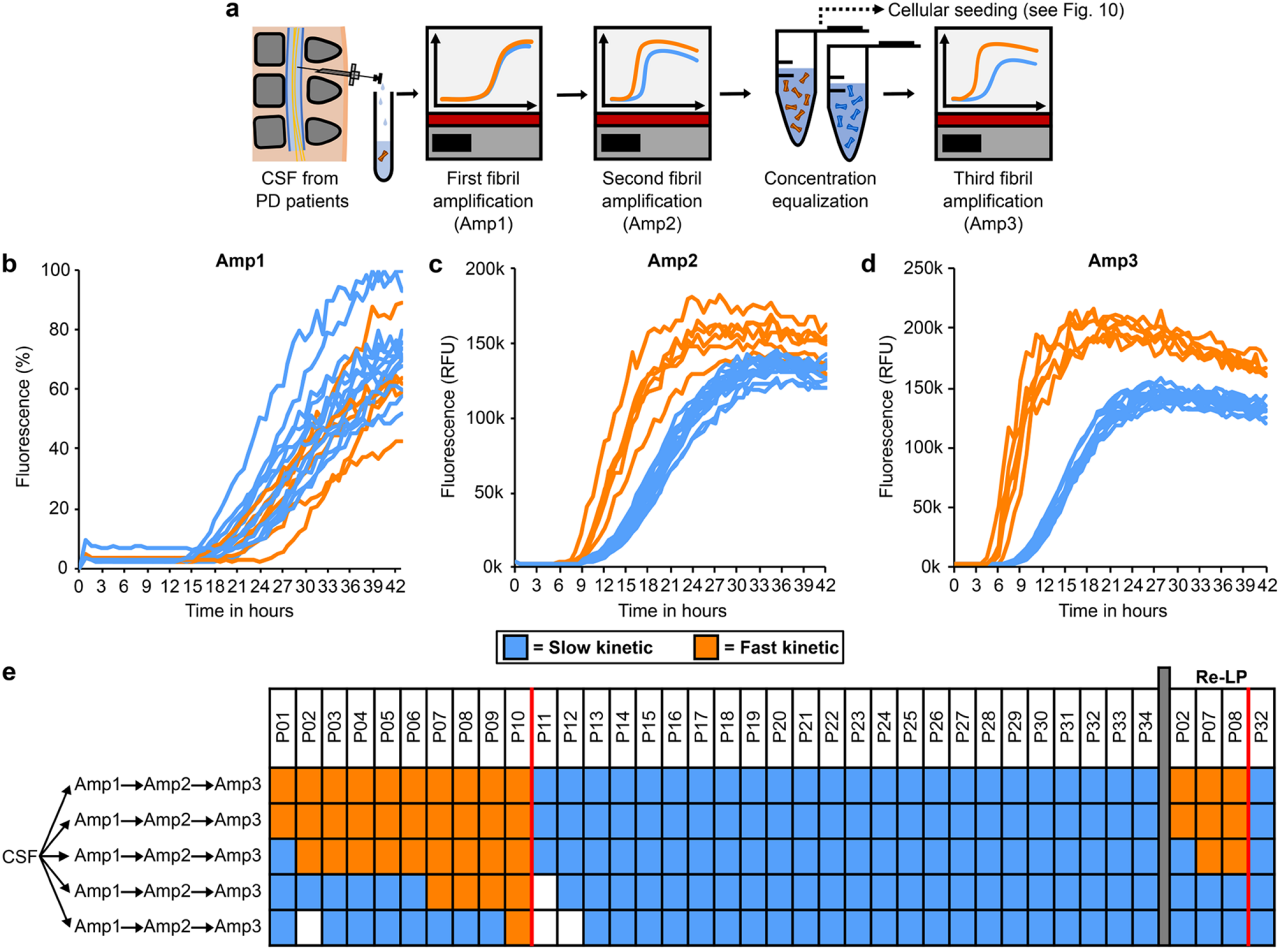
Recursive seed amplification uncovers distinct seeding properties. **a** Schematic of experimental design: Cerebrospinal fluid (CSF) samples from *n* = 34 patients with Parkinson’s disease (PD) were used to seed a seed amplification assay (SAA). The SAA product (Amp1) was diluted and used as seed for another round of amplification (Amp2). Following the second amplification, fibrils were purified, adjusted in concentration and used as seeds for a third SAA (Amp3). **b** SAA aggregation curves of the initial amplification (Amp1) from CSF samples. Each sample was run in four technical replicates. The replicate with the second fastest lag phase is shown. Curves were color-coded based on the kinetic of the last round of amplification (Amp3). **c** SAA aggregation curves in the second amplification (Amp2) using the diluted SAA products from Amp1. Each curve represents the average fluorescence of four independent replicates. **d** SAA aggregation curves in the third amplification (Amp3) using the diluted SAA products from Amp2. Each curve represents the average fluorescence of four independent replicates. Patient samples were classified as “fast kinetic” (orange) or “slow kinetic” (blue) for (b-d) based on this third round of amplification (Amp3). **e** Summary of the kinetic results in Amp3 for each patient as obtained by different experiments. Each panel represents the Amp3 result of the respective patient sample (*n* = 34). For each panel, the seeds were amplified in independent experiments in a new round of CSF-> Amp1-> Amp2-> Amp3 amplification (= rSAA). For four patients, the CSF from a second, independent lumbar puncture (Re-LP) was available. For samples with white panels, not enough CSF was available to perform five independent experiments

In order to detect strains of aSyn pathology, we expanded this classical SAA protocol originating from the Caughey and Parchi groups [16, 40] to several rounds of recursive amplification (Fig. 1a). Specifically, products of a first SAA (Amp1) were harvested, diluted in PBS, and used as seeds for a second round of SAA (Amp2). The products from Amp2 were again harvested and diluted in PBS to an equal amount of 75 pg per well, before being used as seeds for a third and final round of the same SAA protocol (Amp3). In order to discriminate this expanded SAA protocol from the traditional paradigm, we refer to it as “recursive SAA” (rSAA) here. Before measuring the protein concentration, an aliquot of each fibril solution was converted to monomeric protein form using 4 M guanidinium chloride (GdnHCl).

In Amp1, we observed a wide range of aggregation time courses of CSF samples from 34 different patients with PD (Fig. 1b), consistent with previous findings from our group and others [8, 9]. In Amp2, two groups of kinetics began to emerge, characterized by faster and slower aggregation (Fig. 1c). These two groups became more distinct in Amp3 (Fig. 1d). To objectively classify the kinetic type as either fast or slow, at least 15 samples were run on the same plate with additional fibril controls for both kinetic types. Kinetics were classified as fast (orange) and slow (blue) based on the area under the curve (AUC) during an 8-hour interval, starting after the first sample crossed the 10,000 relative fluorescence units (RFU) threshold (illustrated in Supplementary Fig. 1). We observed no systematic difference in the aggregation kinetics during Amp1 when samples were color-coded based on their kinetic type in Amp3 (Fig. 1b). In contrast, color-coding aggregation curves in Amp2 based on the results from Amp3 (Fig. 1c) suggested that seeding properties may be stable, i.e., that the fibril structure underlying the kinetic properties is already present in Amp1 but becomes fully dominant in Amp2 and Amp3 (aggregation curves for individual patients are shown in Supplementary Fig. 3).

To confirm this hypothesis, we performed up to five independent rSAA (CSF-> Amp1-> Amp2-> Amp3) for each of a total of 34 CSF samples and determined the aggregation kinetics in Amp3 as described above (Fig. 1e). For 25 out of 34 CSF samples, the aggregation kinetics in Amp3 were the same in all 3–5 independent rSAA; for 3 samples, aggregation was fast in 4 out of 5 rSAA; for 5 samples, aggregation was fast in 3 out of 5 rSAA; for 1 sample, aggregation was fast in 3 out of 4 rSAA, and for 1 sample, aggregation was fast in 2 out of 5 rSAA. For subsequent analyses, we defined a CSF sample as “fast kinetic” when at least 40% of independent rSAA resulted in fast aggregation kinetics in Amp3 (for details see method section). Importantly, in the four patients for whom CSF samples from a second lumbar puncture were available, identical kinetic seeding properties were observed upon amplification (Fig. 1e, “Re-LP”).

**Fig. 2:**
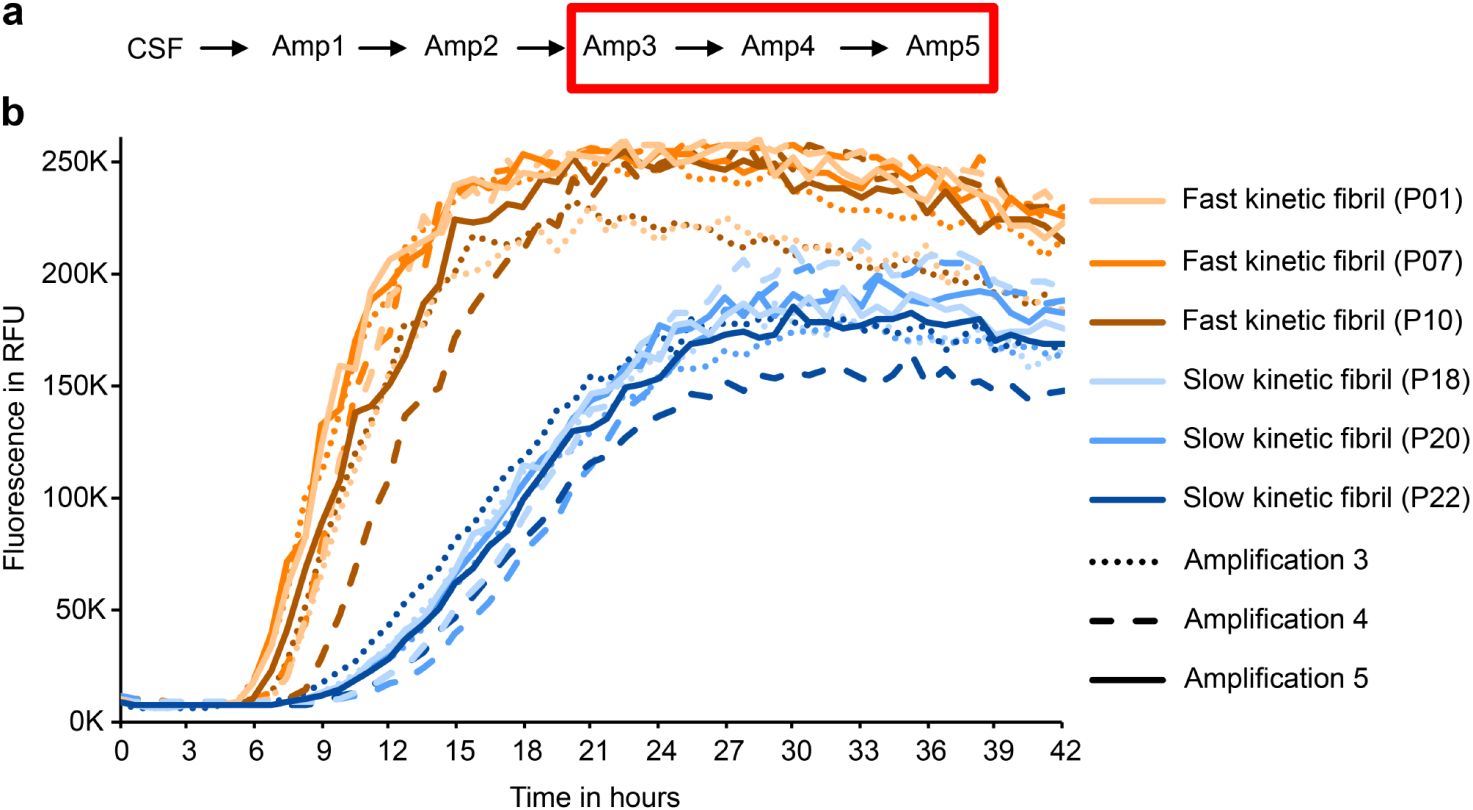
Kinetic types are stable in additional rounds of amplification. **a** Schematic overview of the experiments. **b** Adding additional rounds of amplification (Amp4, Amp5), the kinetic type was unchanged for the analysed samples (P01, P07, P10, P18, P20, P22)

**Fig. 3:**
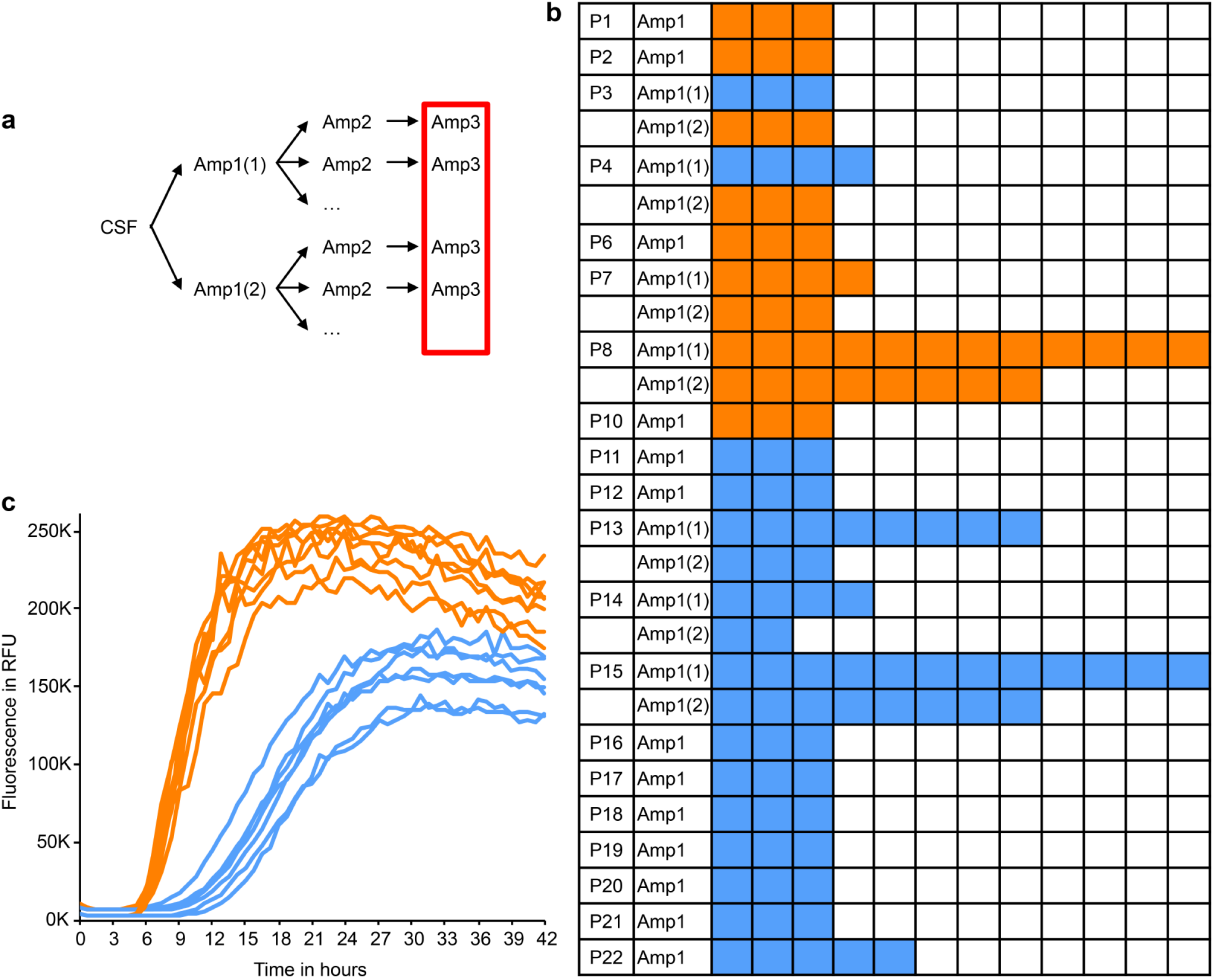
Kinetic types are stable in multiple amplifications from the same Amp1 product. **a** Schematic overview of the experiments. The products of the initial seed amplification from CSF (Amp1) were amplified over several rounds of amplifications (Amp2-> Amp3). **b** Each panel represents the results of one round of amplification (Amp2-> Amp3 (orange = fast kinetic; blue = slow kinetic; white = not done). Each row represents amplifications from the same Amp1 product. **c** Curves depict Amp3 results of several amplifications of the Amp1(1) product from sample P8 (orange) and sample P15 (blue)

To confirm the stability of seeding properties, Amp3 products were amplified in additional rounds of Amp4-> Amp5, showing stable kinetic properties (Fig. 2). To explore the emergence of the two fibril types in more detail, we amplified products from Amp1 for up to 12 rounds of Amp2-> Amp3 and classified the aggregation kinetics in Amp3 as described above. For each Amp1 product, the same kinetic behavior was observed in every independent round of Amp2-> Amp3 amplification (Fig. 3). This finding confirms that the fast vs. slow kinetic type is a stable property of SAA products and suggests that the kinetic type in Amp3 is defined by the product of Amp1. Taken together, these findings suggest that the kinetic type of fibrils in Amp3 originates from conformational differences of aSyn seeds in patient CSF.

### Fibril types are not dictated by CSF components other than seeds and are stable against interim changes in buffer conditions

**Fig. 4:**
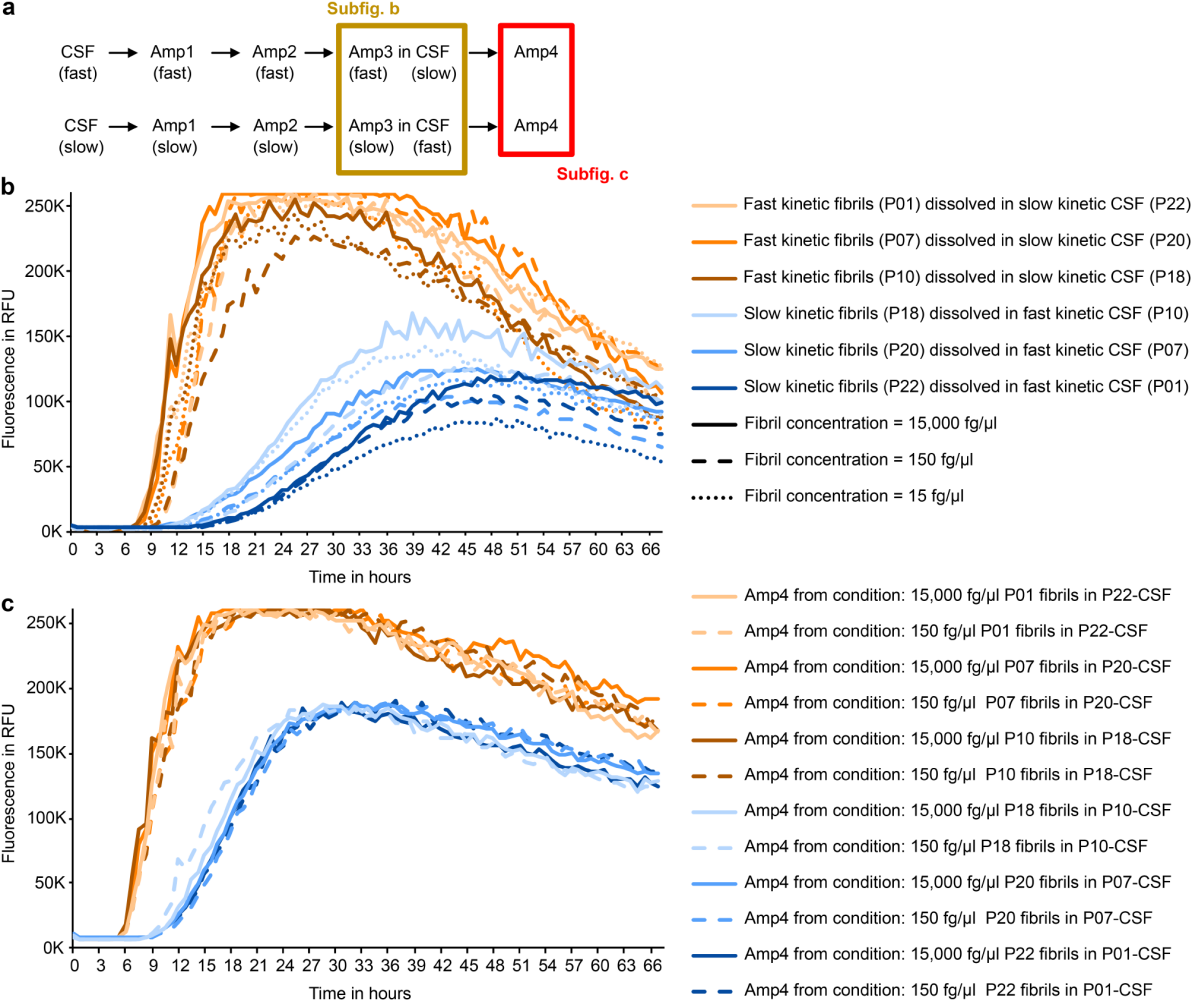
Kinetic types are unaffected by donor CSF components other than seeds. **a** Schematic overview of the experiments. In addition, fibrils from Amp1 were separately amplified as described in Fig. 1 to test their kinetic type. CSF (slow) = CSF from patients defined as slow kinetic sample; CSF (fast) = CSF from patients defined as fast kinetic sample; AmpX (slow) = slow kinetic fibrils; AmpX (fast) = fast kinetic fibrils. **b** Figure shows the third amplification (Amp3) of aggregates from three fast kinetic samples (P01, P07, P10) dissolved in CSF from slow kinetic samples (P18, P20, P22) (light orange, orange and dark orange curves) and vice versa (light blue, blue and dark blue curves). For each sample, 3 different dilutions of aggregates were tested (15 fg/µl = dotted line; 150 fg/µl = dashed line; 15,000 fg/µl = solid line). The kinetic type was not influenced by the CSF under these conditions. **c** Figure shows the fourth amplification (Amp4), here without CSF, with the same dilution of Amp3 products (1:100,000). The fibrils preserved their initial kinetic type regardless of the CSF or the amount of seeds added in Amp3

Kinetic features of SAA may be influenced by CSF components [4]. To determine whether this is the case for the rSAA kinetic type, we created hybrid samples by combining (a) products from Amp2 that show fast kinetic behavior in Amp3 and (b) CSF of samples with slow kinetic behavior in Amp3 – and vice versa. When these hybrid samples were used as seeds for the SAA, the kinetic type was determined by the type of rSAA product, and not by the type of CSF (Fig. 4).

It is noteworthy that in these experiments with hybrid samples, the CSF was not depleted of aSyn protein, so they still contained the “original” aSyn seeds. To minimize the effect of these “original” seeds, Amp2 fibrils were added at concentrations down to 15 fg/µl. This is probably still higher than the concentration of the “original” seeds, which has been estimated as 1 to 0.1 fg/µl in the final mix [16]. Yet, this finding is consistent with the hypothesis that the rSAA kinetic type results from the aSyn seeds, and not from components or environmental conditions of the CSF.

The structure of aSyn fibrils that are amplified from a synthetic seed can be dictated by assay conditions [33]. To confirm that in rSAA, the amplified fibril type is determined by the seeds and not by the assay conditions, we interposed an amplification of the two fibril types in several modified SAA protocols. In these experiments, the kinetic type was preserved when – in an interposed amplification round – buffers, salts, and pH were changed and SDS was removed (Supplementary Fig. 4b). Moreover, stable kinetic types were observed with 4 different batches of aSyn monomer - provided that monomer meeting quality criteria was used (see method section).

### Neither unseeded aggregation nor differing seed concentrations explain fast and slow kinetic types

**Fig. 5:**
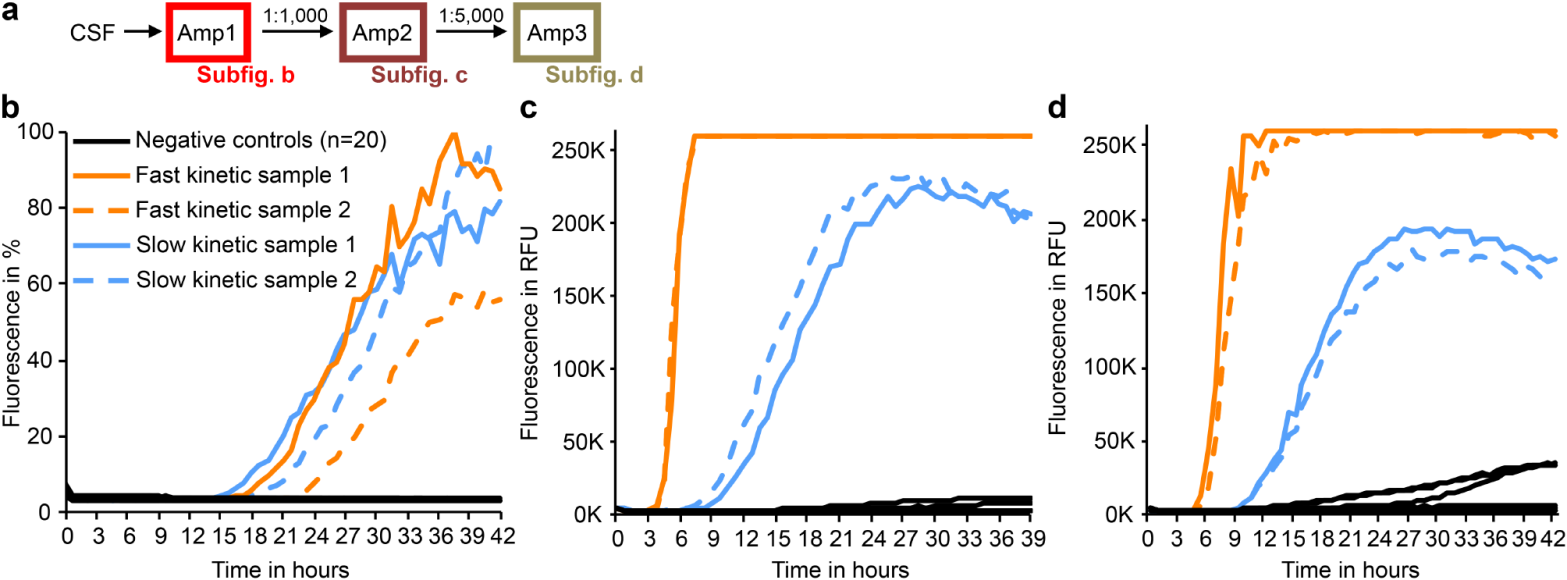
Unseeded aggregation curves in negative controls does not resemble fast or slow kinetic. **a** Schematic overview of the experiments. **b** SAA aggregation curves of the initial amplification (Amp1) from CSF. 24 samples were measured on the same plate, each sample was run in four technical replicates. 20 samples were previously measured negative in the SAA (black curves), 2 PD-samples were previously categorized as fast kinetic (orange curves) and 2 PD-samples were previously categorized as slow kinetic (blue curves). The replicate with the second fastest lag phase is shown. **c** SAA aggregation curves in the second amplification (Amp2) using the diluted SAA products from Amp1. Each curve represents the average fluorescence of four independent replicates. The kinetic type of the 4 PD samples is already distinguishable. Two negative controls show a small increase in the fluorescence signal. **d** SAA aggregation curves in the third amplification (Amp3) using the diluted SAA products from Amp2. Each curve represents the average fluorescence of four independent replicates. The kinetic type of the 4 PD samples is distinguishable. Three of the negative samples show an increase in the fluorescence signal, two of them already did in Amp2

To minimize the effects of random aggregation events, the entire rSAA procedure was conducted independently at least 5 times per sample, given that enough CSF was available (Fig. 1e). To rule out any contribution of unseeded aggregation, we performed rSAA with CSF from 20 negative controls and 4 PD patients. Indeed, unseeded aggregation was observed in 2 of 20 negative controls in Amp2 (Fig. 5). When the products were used as seeds for Amp3, 3 of 20 controls showed aggregation. However, the observed kinetic was clearly distinct from the products of PD samples that were amplified on the same plate. This finding demonstrates that unseeded aggregation does not contribute to the fast and slow kinetic fibrils observed with PD samples. In addition, it suggests that fibrils seeded with CSF from patients with PD are biophysically distinct from fibrils obtained by unseeded aggregation.

Reactions seeded with SAA products showed a much shorter lag phase than unseeded reactions (Fig. 5c). We assume that the aggregation process is dominated by the most rapid reaction, so when seeds such as SAA products are present, unseeded aggregation might not occur.

**Fig. 6:**
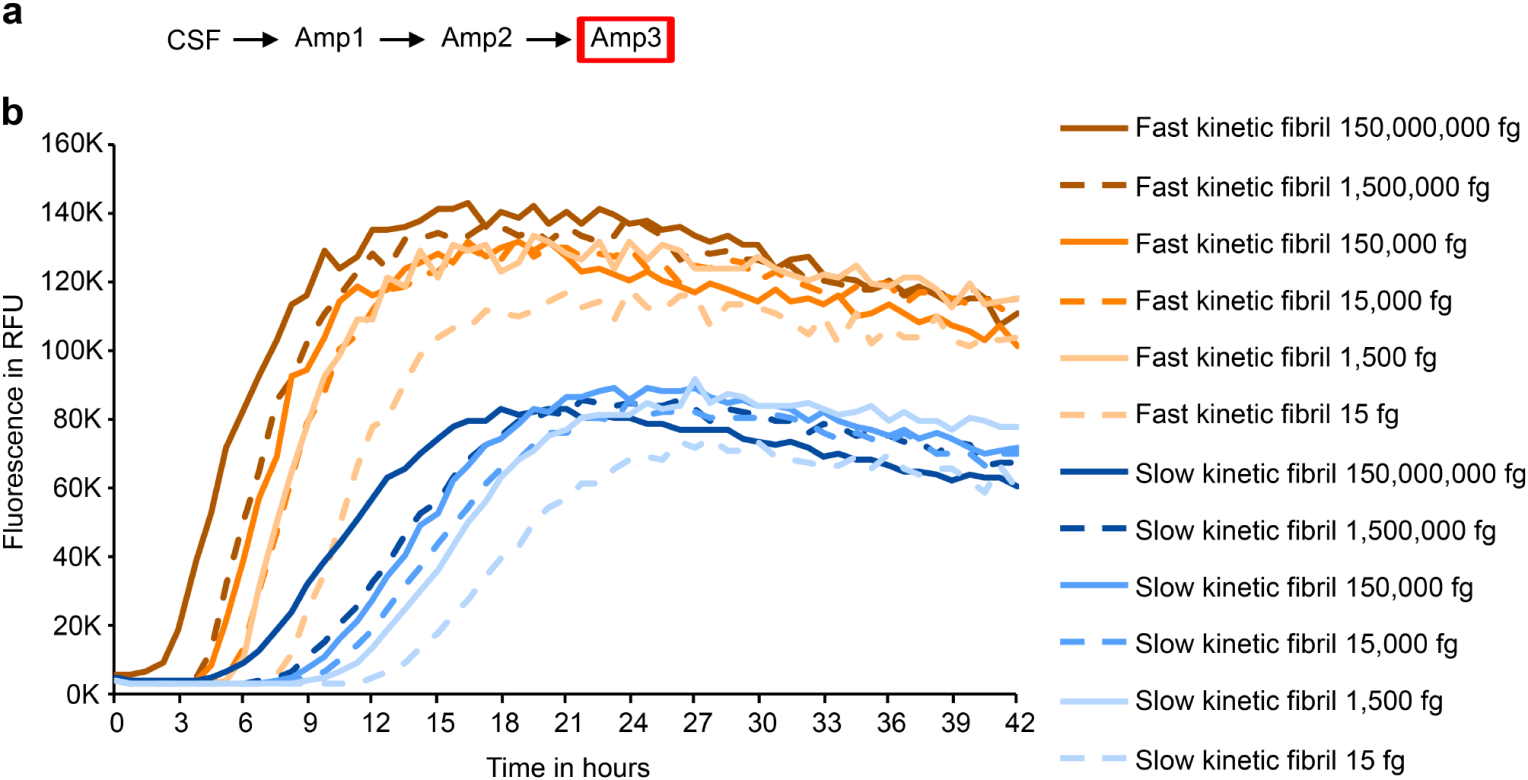
Distinguishability of fibril types is preserved despite differing fibril concentrations. **a** Schematic overview of the experiments. In addition, fibrils from Amp1 were separately amplified as described in Fig. 1 to test their kinetic type. **b** The third round of amplification (Amp3) was seeded with different amounts of fast (light orange, orange and dark orange curves) and slow kinetic fibrils (light blue, blue and dark blue curves). Each curve represents the average of four independent replicates

We assume that the concentration of seeds in Amp2 and Amp3 is several folds higher than in Amp1, allowing the aggregation reaction to be dominated by the type of aSyn fibril. In addition, the same amount of Amp2 products was used to seed Amp3. Still, we could not rule out entirely that aggregation kinetics in Amp2 and Amp3 were affected by the concentration of aSyn seeds. Therefore, we investigated the effect of fibril concentration on the kinetic properties of the aggregation reaction. Even a 1,000-fold difference in seed concentration did not alter the kinetic type (Fig. 6) as defined in the method section (and Supplementary Fig. 1).

Irrespective of the concentration used, the lag phase of fast kinetic fibrils was shorter than the lag phase of slow kinetic fibrils (Fig. 6 and Supplementary Fig. 5). The only exception was the lag phase of a very high concentration of slow kinetic fibrils (150,000,000 fg) (lag phase = 6.6 h). It was in a similar range as the lag phase of fast kinetic fibrils (lag phase (fast kinetic fibril 1,500 fg) = 6.4 h) and even shorter compared to the seven orders of magnitude lower concentration of fast kinetic fibrils (15 fg) (lag phase = 8.25 h). Even though the lag phase was similar for these curves (6.4 h (fast kinetic fibril 1,500 fg) vs. 6.6 h (slow kinetic fibril 150,000,000 fg); *P* = 0.99, two-way ANOVA with Tukey´s multiple comparison test), the slope (308.7 RFU/min (fast kinetic fibril 1,500 fg) vs. 132.7 RFU/min (slow kinetic fibril 150,000,000 fg); *P* = 0.0003, two-way ANOVA with Tukey´s multiple comparison test) and maximum fluorescence (133032.8 RFU (fast kinetic fibril 1,500 fg) vs. 81934 RFU (slow kinetic fibril 150,000,000 fg); *P* < 0.0001, two-way ANOVA with Tukey´s multiple comparison test) was still significantly different (Fig. 6). The concentration of seeds thus may impact the lag phase, but shows only minor effects on other kinetic parameters (see Supplementary Fig. 5 for more details). We therefore conclude that changes in the fibril concentration cannot explain the kinetic types observed in rSAA from PD samples.

### A mixture of fibrils in CSF might underlie the emergence of kinetic differences

As noted above, the aggregation kinetics become increasingly different between individual CSF samples from Amp1 to Amp2 and from Amp2 to Amp3 (Fig. 1b-d) without further changes in subsequent amplification rounds (Fig. 2). This observation cannot be explained by the dilution of CSF over the course of rSAA because the kinetic type was unaltered when fibrils were diluted in CSF, as noted above (Fig. 4).

Alternatively, a mixture of fibril types, potentially even in the initial CSF, might underlie the emergence of kinetic differences in rSAA. In SAA, the amount of aggregated aSyn can increase by elongation + fragmentation of fibrils and by secondary nucleation + elongation. Fibril structure and SAA conditions can affect which of the two processes predominates [10]. Hence, an SAA protocol can favor the amplification of certain types of fibrils. Therefore, the existence of a variety of different aSyn seeds (strains) in the CSF samples could explain the emergence of clear kinetic differences over the course of the rSAA (Fig. 1b-d). Moreover, the assumption of a mixture of seeds in the CSF could explain why the fast kinetic behavior was not observed in every round of rSAA for CSF from patients P1-P9 (Fig. 1e). Indeed, the ratio of fast kinetic to slow kinetic fibrils observed in rounds of rSAA could relate to the relative abundance of different fibril types, potentially even in patient CSF.

**Fig. 7:**
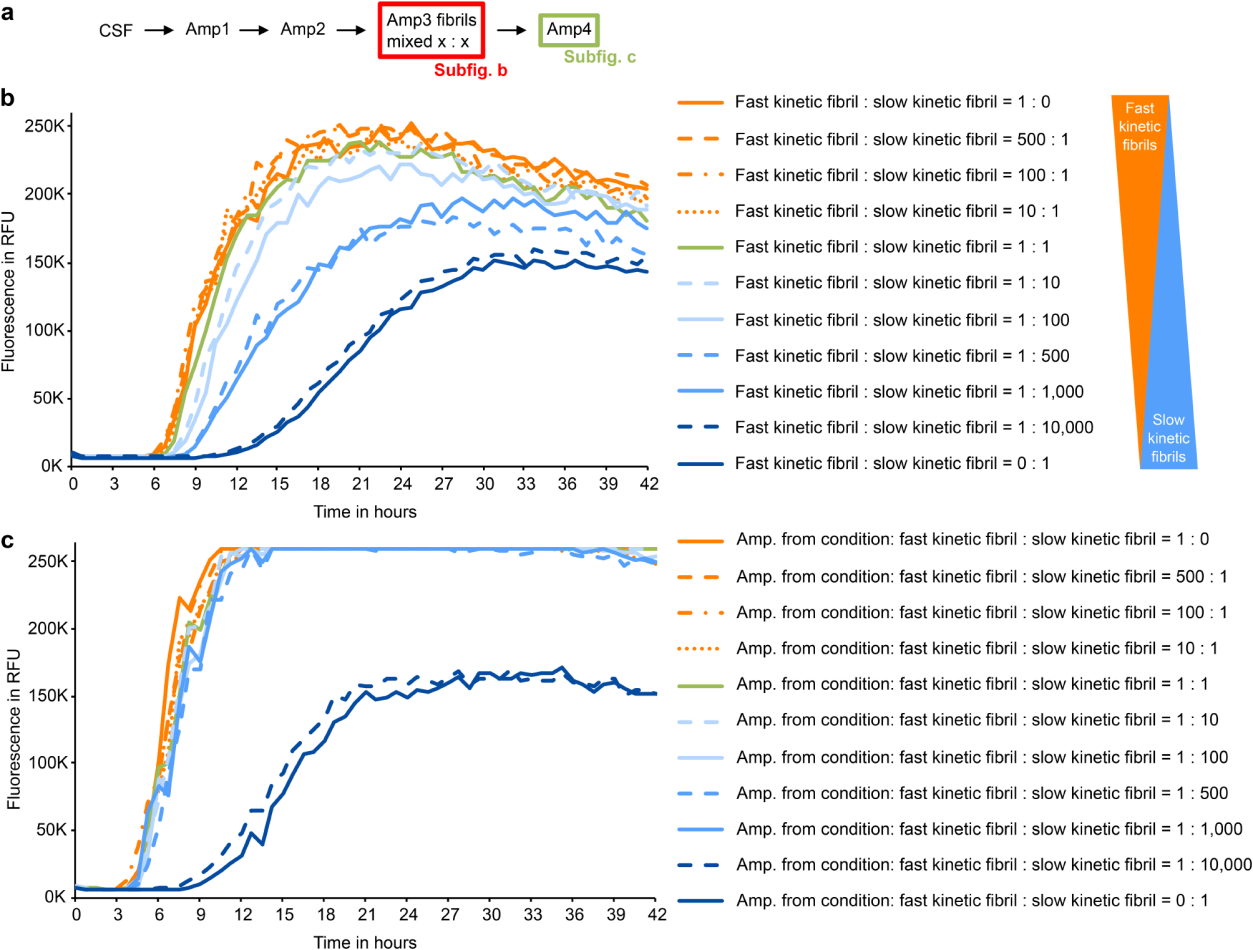
Curve kinetics in fibril mixtures are dominated by fast kinetic fibrils. **a** Schematic overview of the experiments. In addition, fibrils from Amp1 were separately amplified as described in Fig. 1 to test their kinetic type. **b** The third round of amplification (Amp3) was seeded with 75 mg of a fibril mixture, containing different proportions of fast and slow kinetic fibrils. Each curve represents the average of four independent replicates. **c** Figure shows the fourth amplification (Amp4), diluted Amp3 products (1:10,000) were used as seeds. Each curve represents the average of four independent replicates

To support this hypothesis, we used mixtures of slow and fast kinetic fibrils as seeds (Fig. 7). At equal concentrations, fast kinetic fibrils dominated the amplification reaction, showing no difference from undiluted fast kinetic fibrils (Fig. 7). At proportions of fast: slow = 1:10 and 1:100, the lag phase was just slightly longer. At fast: slow = 1:500 and 1:1000 the aggregation curves showed an intermediate kinetic. Only when slow kinetic fibrils predominated by 10,000 fold (fast: slow = 1:10,000), the aggregation kinetic was similar to seeding with undiluted slow kinetic fibrils. When the products of these reactions were used as seeds for a further SAA (Fig. 7c), intermediate kinetics were not observed and two clearly distinct kinetic types reemerged. We propose that the intermediate kinetics observed in Fig. 7 constitute a model for the intermediate kinetics observed in Amp2, whereas the dominance of one kinetic fibril type in Fig. 7 constitutes a model for the dominance of one kinetic fibril in Amp3. The fact that there are no kinetic changes in additional rounds of amplification (Fig. 2) further supports this hypothesis. In fact, this finding indicates that fast and slow kinetic fibrils might not be the only fibrils present in patient CSF.

### The phenotype of random fibrils is distinct from fast kinetic and slow kinetic fibrils

**Fig. 8:**
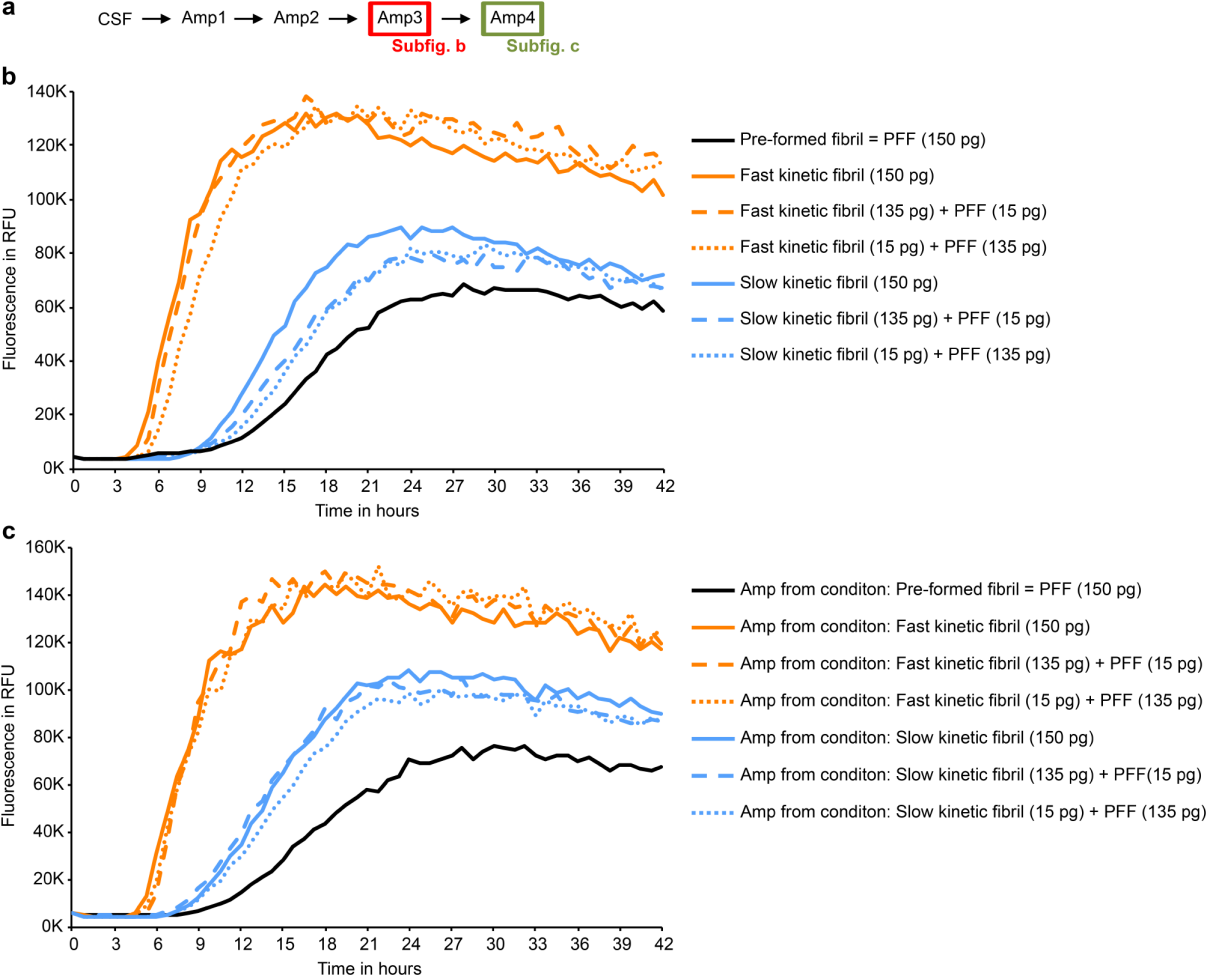
Unseeded aSyn pre-formed fibrils show a different kinetic type in rSAA compared to fast and slow kinetic fibrils. **a** Schematic overview of the experiments. In addition, fibrils from Amp1 were separately amplified as described in Fig. 1 to test their kinetic type. **b** The third round of amplification (Amp3) was seeded with 150 pg of either fast kinetic fibrils (orange line), slow kinetic fibrils (blue line), pre-formed fibrils (PFF) (black line) or a mixture of either fast or slow kinetic fibrils with PFF (dashed and dotted lines). Each curve represents the average of four independent replicates. PFF show a distinct kinetic curve (slope; maximum fluorescence) compared to fast and slow kinetic fibrils. **c** Figure shows the fourth amplification (Amp4), diluted Amp3 products (1:5,000) were used as seeds. Each curve represents the average of four independent replicates. The kinetic differences between PFF, fast kinetic and slow kinetic fibrils are still present

Recent cellular and animal PD models use aSyn pre-formed fibrils (PFF), which are generated by unseeded aggregation of aSyn monomer [36]. Because the kinetic behavior of fibrils emerging in negative controls was quite distinct (Fig. 5), we compared the kinetic behavior of fibrils obtained from patient CSF by rSAA to PFF. Indeed, SAA kinetics seeded by PFF were quite distinct from SAA kinetics seeded by fast and slow kinetic fibrils (Fig. 8b). PFFs showed an even longer lag phase (11.25 h vs. 9 h), lower maximal fluorescence (74244 RFU vs. 106566.8 RFU) and smaller slope (71.8 RFU/min vs. 133.9 RFU/min) than slow kinetic fibrils. These differences were stable over multiple rounds of amplification (Fig. 8c). This demonstrates that the rSAA is able to amplify more than just two distinct kinetic types. Fast kinetic fibrils were less affected by mixing with PFF as seeds showed a just slightly longer lag phase. In contrast, SAA with a mixture of PFF with slow kinetic fibrils showed intermediate kinetics (Fig. 8b). Importantly, the influence of PFF was no longer detectable when reaction products were used as seeds for another round of SAA (Fig. 8c). This is consistent with our observations with mixtures of fast and slow kinetic fibrils (Fig. 7c) and with the concept that the seed with the fastest aggregation kinetic generally dominates the SAA.

### Modified conformational stability assay confirms distinct biophysical properties of aSyn fibril types

**Fig. 9:**
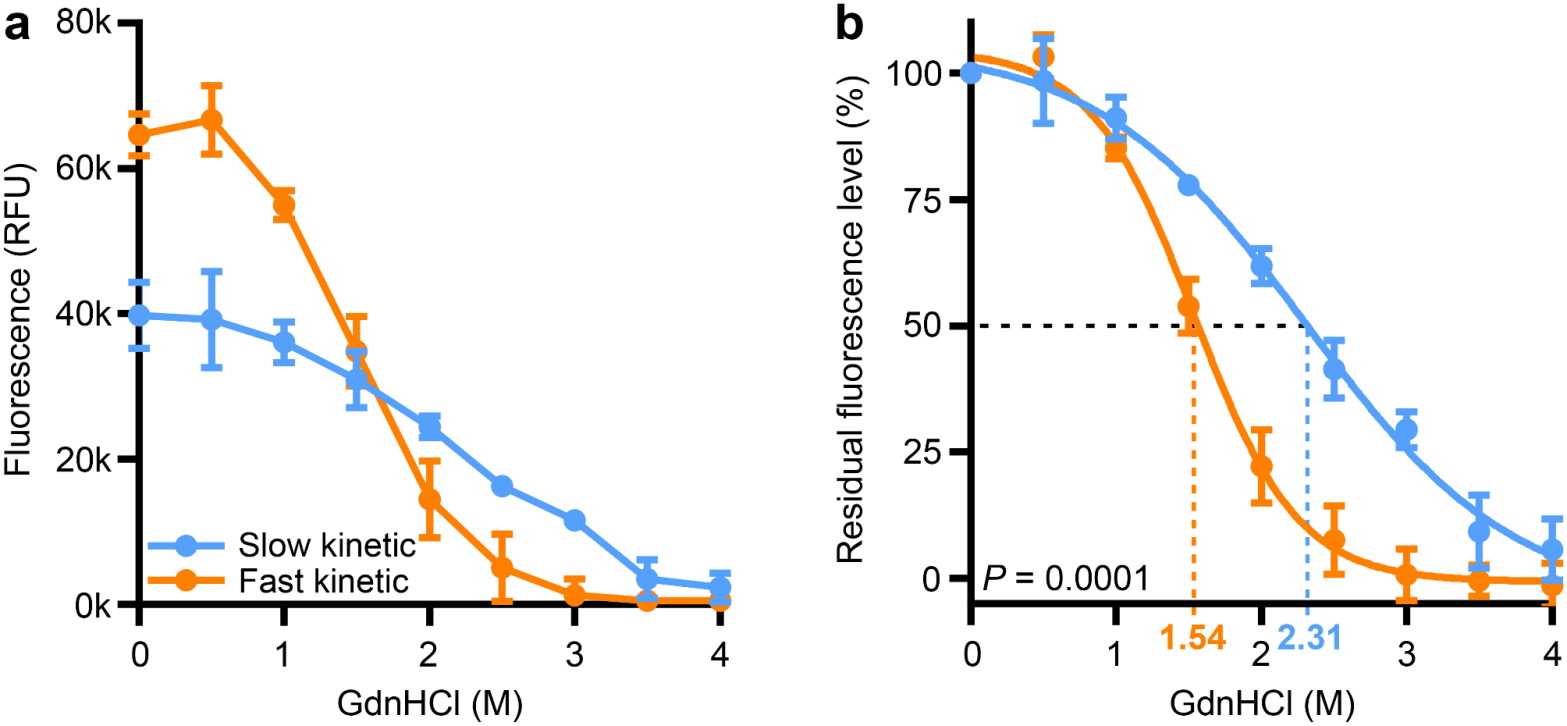
Fast and slow kinetic fibrils differ in their conformational stability. Comparison of fibrils with fast versus slow recursive seed amplification assay (rSAA) aggregation kinetics regarding their resistance to increasing concentrations of the chemical denaturant guanidinium chloride (GdnHCl). **a** Raw fluorescence values (minus blank control) after incubation with thioflavin T show a lower baseline (0 M GdnHCl) fluorescence of slow kinetic fibrils (*P* = 0.0014). **b** Non-linear curve fit of fluorescence values normalized to baseline shows that slow kinetic fibrils have a higher GdnHCl_50_ value, i.e., the concentration of GdnHCl required to denature 50% of the fibrils (2.31 M vs. 1.54 M, *P* = 0.0001). Values are mean ± standard deviation (*n* = 3). *P* values from two-tailed unpaired *t* test. RFU = Relative fluorescence units

To further characterize the fibrils, we determined the stability of SAA products against the chaotropic agent GdnHCl in a modified conformational stability assay (CSA) [26]. Instead of employing a Western blot, we used ThT fluorescence as a readout to detect the remaining aggregates unaffected by GdnHCl. When comparing equal amounts of the two fibril types, slow kinetic fibrils exhibited a significantly lower baseline fluorescence in the 0 M GdnHCl condition (*P* = 0.0014; Fig. 9a). This indicates that ThT interacts differently with the two fibril types, supporting the hypothesis of structural differences. Furthermore, slow kinetic fibrils showed a significantly higher resistance to chemical denaturation, as indicated by a higher GdnHCl concentration required to solubilize 50% of the fibrils (GdnHCl_50_ 2.31 M vs. 1.54 M, *P* = 0.0001; Fig. 9b). These distinct biophysical properties can potentially be explained by a more compact structure of the slow kinetic fibrils, which might interfere with intercalation of ThT and denaturation by GdnHCl.

### Patient-derived fibrils differ in their capacity to induce cellular aSyn pathology

**Fig. 10:**
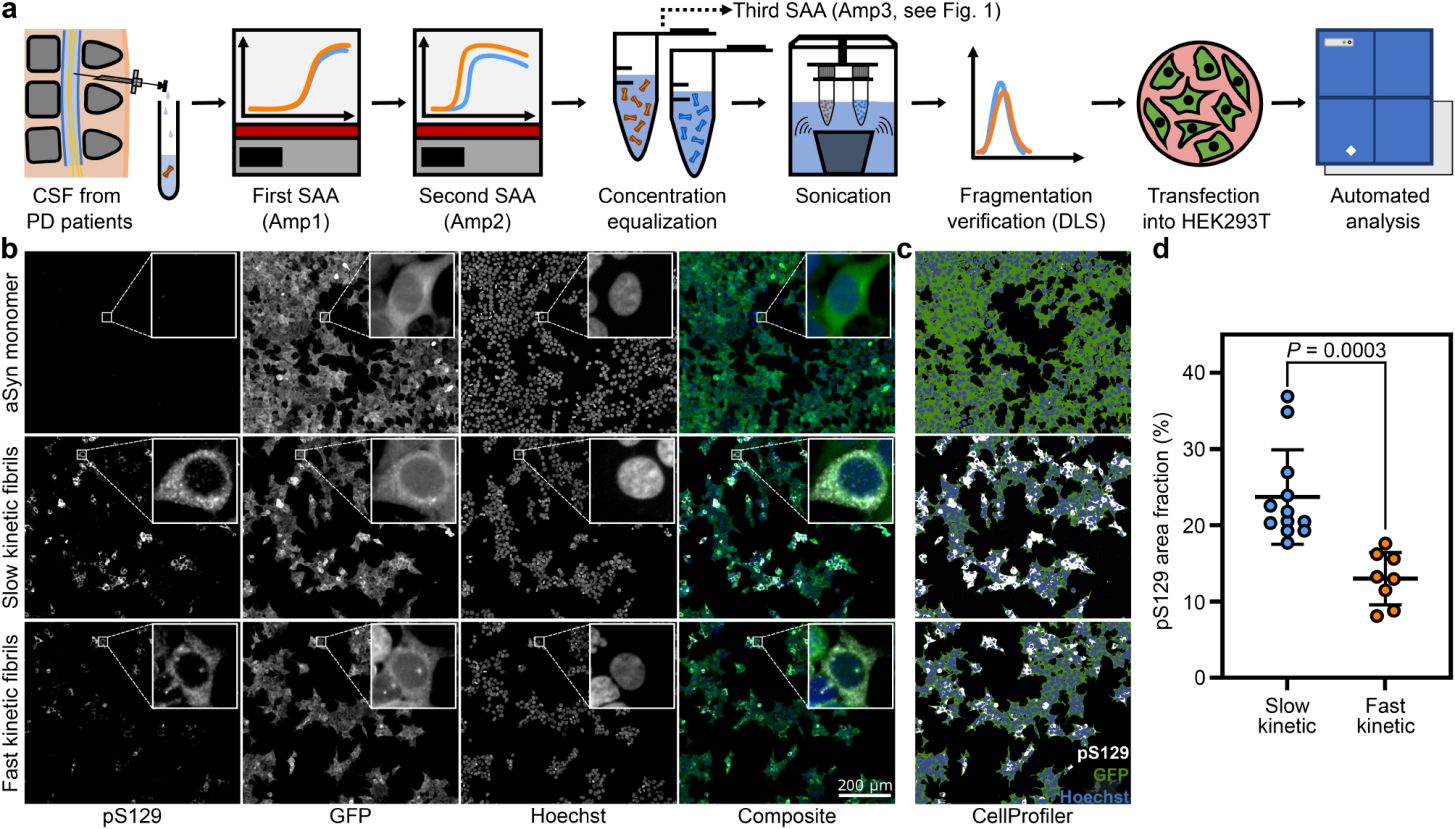
Patient-derived fibrils differ in their capacity to induce cellular α-synuclein pathology. **a** Schematic of experimental design: α-synuclein (aSyn) seeds present in cerebrospinal fluid (CSF) samples from patients with Parkinson’s disease (PD, *n* = 20) were amplified twice using a seed amplification assay (SAA). Following amplification, fibrils were purified, adjusted in concentration, and sonicated. Successful and comparable fragmentation was verified by dynamic light scattering (DLS). The sonicated fibrils were subsequently transfected into three HEK cell clones stably overexpressing both aSyn and GFP separately (3 wells per patient sample, clone, and experiment). After incubating for 48 h, the cells underwent fixation, staining, and automated imaging. **b** Representative immunofluorescence images show staining for phosphorylated aSyn (pS129), GFP, and Hoechst of cells treated with aSyn monomer (control) and patient-derived fibrils (fast kinetic = Patient 01, slow kinetic = Patient 21). **c** Thresholding results of images from (b) after automated analysis using CellProfiler. Regions of interest were obtained for areas positive for pS129 (white) and GFP (green). **d** pS129 area fraction (percentage of GFP-positive cytosol covered by pS129 signal) after transfection with patient-derived fibrils with fast (*n* = 8) versus slow (*n* = 12) rSAA kinetics. Each dot represents the mean of all experiments (*n* = 3–8, see Supplementary Table 2) of one patient in all three HEK cell clone. Lines represent mean ± standard deviation, *P* value from two-tailed unpaired *t* test

To determine whether the biophysical differences of the fast and the slow kinetic fibril types are also biologically relevant, we assessed their capacity to seed aSyn pathology in cells. As rSAA products are templated by aSyn seeds in the CSF of patients, we refer to them as “patient-derived fibrils” (PDFs) here. PDFs from 20 patients were obtained from CSF after two rounds of amplification, adjusted to equal protein concentrations, sonicated, and transfected into HEK293T cells stably overexpressing aSyn and GFP separately (Fig. 10a). Sonication was performed to facilitate fibril uptake into cells and to eliminate potential differences in fibril size; fragmentation was confirmed by dynamic light scattering in each experiment (Supplementary Fig. 6a + b). Insufficiently fragmented fibrils were excluded. Cells were fixed 48 h after transfection. The extent of aSyn pathology was quantified by staining for aSyn phosphorylated at serine 129 (pS129, Fig. 10b), the most common post-translational modification in Lewy pathology [2, 15]. As the fibrils are modified inside of the cell, pS129 resembles biologically relevant pathology.

PDFs were transfected into three separate clones of HEK293T cells across 3–8 independent experiments, with three technical replicates performed per experiment. This resulted in over 150,000 cells imaged per patient sample (Supplementary Table 2). Using automated image analysis, the GFP-positive cytoplasm was detected, and the area fraction positive for pS129 was quantified (Fig. 10c). PDFs with slow rSAA kinetics demonstrated a significantly greater capacity to induce phosphorylated aSyn pathology compared to those with fast rSAA kinetics, as evidenced by both a higher pS129 area fraction and a greater percentage of pS129-positive cells (Fig. 10d, Supplementary Fig. 6c). After 48 h, the number of cells per well did not differ between both fibril types, indicating no significant difference regarding cell proliferation or death (Supplementary Fig. 6d). Together, these findings show that the two fibril types differ in their biological properties. The variability in the cellular seeding capacity between different PDFs of one fibril type may reflect further conformational heterogeneity that is not detected in rSAA kinetics.

#### Seeding behavior is associated with clinical features

We previously described faster aggregation in SAA, using CSF from patients with more severe motor and cognitive impairment [8]. Specifically, we observed a correlation between SAA lag phase and clinical scores for cognitive and motor function - without observing two qualitatively different types of aggregation kinetics, consistent with our current findings in Amp1 (Fig. 1b). Given that aSyn pathology is more widespread in patients with more severe motor and cognitive impairment [22, 27], the shorter lag phase in the CSF of these patients is most likely caused by a higher number of aSyn seeds. The rSAA was designed to mitigate the effect of the very low number of seeds and other factors in the initial CSF by harvesting fibrils after reaching the plateau phase of seed amplification, by dilution and by adjusting the fibril concentration (Fig. 1a). We were therefore curious to see which clinical features in patients were associated with each of the two kinetic types of fibrils amplified from CSF by rSAA.

In patients with only slow kinetic type in rSAA, postural instability and gait difficulty (PIGD) motor symptoms and REM sleep behavior disorder (RBD) were significantly more common than in patients with a fast kinetic type in rSAA (Table 1). There was also a trend towards a higher prevalence of depression and sleep disturbance in patients with only slow kinetic type in rSAA, although this difference was not statistically significant. Because the motor phenotype can change over time, and differences between clinical phenotypes in PD tend to decrease over the course of the disease [1, 35, 53], we only analyzed non-motor symptoms during the first 3 years after motor onset. While our findings need to be confirmed in larger cohorts, they suggest that some of the clinical heterogeneity in PD [13, 18, 20, 24] may be explained by the presence of strains, that lead to different aSyn fibril types as reported by the rSAA.

**Table 1:** Clinical characteristics of CSF donors. ^a^Fisher’s exact test, P values adjusted using the Holm-Ŝidák method. ^b^Parameters: fast kinetic PIGD, fast kinetic non-PIGD, slow kinetic PIGD, slow kinetic non-PIGD. ^c^Parameters: fast kinetic “yes”, fast kinetic “no”, slow kinetic “yes”, slow kinetic “no”. PIGD: postural instability and gait difficulty, TD: Tremor dominant, RBD: Rapid eye movement sleep behavior disorder

Clinical features (first 3 years of disease)	Fast kinetic	Slow kinetic	*P* value^a^
Motor subtype (PIGD/intermediate/TD/unknown)	1/2/7/0	16/4/3/1	**0.014** ^b^
RBD (yes/no/unknown)	1/9/0	12/6/6	**0.029** ^**c**^
Depression (yes/no/unknown)	2/7/1	11/9/4	0.34^c^
Constipation (yes/no/unknown)	2/7/1	8/12/4	0.5^c^
Hyposmia (yes/no/unknown)	4/3/3	16/3/5	0.5^c^
Sleep disturbance (yes/no/unknown)	2/7/1	13/6/5	0.15^c^

## Discussion

In this study, CSF from patients with PD was used for recursive seed amplification (rSAA). With this straightforward principle, two types of fibrils emerged, characterized by fast and slow aggregation kinetics (Fig. 1d). This property likely emerged in the first round of amplification (Amp1, Fig. 3), was stable over multiple rounds of amplification (Fig. 2) and remarkably predictable when CSF from the same patient was used as template (Fig. 1e). The fibril type was also stable when interposing different SAA conditions (Supplementary Fig. 4) and unaffected by other CSF factors (Fig. 4). Neither differences in fibril concentration nor unseeded aggregation can explain the two kinetic types (Fig. 5 + 6 + 8). Furthermore, the slow kinetic and fast kinetic fibril types differed in their resistance to GdnHCl and their interaction with ThT in a modified conformational stability assay (Fig. 9). From these findings, we conclude that the two fibril types amplified by rSAA differ in their aSyn conformation.

These conformational differences are biologically relevant, shown by the fact that slow kinetic fibrils were more effective at seeding phosphorylated aSyn pathology in cells than fast kinetic fibrils (Fig. 10). Furthermore, fibrils with a slow kinetic in the rSAA and higher cellular seeding capacities were predominantly amplified from CSF of patients with a PIGD motor phenotype and RBD (Table 1). Therefore, we interpret our findings as evidence for different strains of aSyn pathology, present in patients with PD.

The general existence of aSyn strains has already been reported in previous studies. Different strains of artificially generated fibrils have been described [7, 17, 34]. Furthermore, fibrils found in different disease entities have been linked to aSyn strains. It is not clear whether the different fibrils are the primary cause of different disease phenotypes, or rather a result of different environments generated during the course of disease. Nonetheless, distinct strains have been associated with PD, a neuronal synuclein disease, and MSA with predominantly glial aSyn pathology. For instance, SAA protocols using CSF can differentiate between patients with PD and patients with MSA [41]. Furthermore, SAA products from patients with PD and MSA can induce distinct intracellular inclusions in HEK293 cells without kinetic differences in the initial SAA [29], and SAA products can differ in their proteolytic stability and neuronal toxicity [51]. Similarly, brain homogenates derived from patients with PD and MSA differ in their capacity to induce neuropathology and motor deficits in rats [52].

In contrast to these previous studies, we describe here a heterogeneity of aSyn pathology - as reported by the rSAA - within the PD entity. Consistent with these experimental findings, several PD subtypes have been proposed based on initial symptoms and the course of disease progression in PD (e.g., PIGD vs. TD; diffuse-malignant vs. mild motor-predominant) [13, 18, 24]. Moreover, two subtypes of PD have been proposed based on the presumed anatomical location of the initial aSyn aggregation (brain-first vs. body-first) [6, 20]. Further analyses correlating the kinetic fibril type with clinical features and neuropathological distribution in larger and well-defined cohorts are required to substantiate the hypothesis that aSyn strains are a possible origin of these subtypes. This is especially important for the association with clinical subtypes such as PIGD vs. TD which are not always constant over time [53]. Even if such subtypes could be influenced by aSyn strains, further factors likely contribute to the clinical phenotype as the disease progresses. For example, two aSyn strains could differ in their capacity to seed further aggregates. The strain with the higher seeding capacity could therefore cause a higher aggregate load and impact cognitive performance earlier. However, the strain with the lower seeding capacity could cause the same aggregate burden, only at a later time point, still resulting in cognitive impairment. Additionally, factors such as beta-amyloid pathology will have an aSyn-strain-independent effect on cognitive impairment.

There have been heterogeneous results considering the products of seed amplification assays. Some differences seemed to be the result of random effects or assay conditions [33], others were shown to be reproducible products of disease strains [41]. However, as we extensively demonstrate in our study, the structure of the fibrils we generate via rSAA is not affected by assay conditions other than seeds. The association with clinical symptoms further supports the notion that the kinetic differences result from differences in the aSyn seeds contained in patient CSF – as expected for aSyn strains. Furthermore, the capacity to detect different strains by amplifying different assay products has been documented for the RT-QuIC (“Real Time Quaking Induced Conversion”) version of the SAA we used here [30].

We found that conformations that form fibrils more rapidly in SAA are associated with less aSyn pathology in cells (Fig. 10d) and with a more benign phenotype in patients (Table 1). This counterintuitive observation could potentially be explained by a more compact structure of the slow kinetic fibrils as suggested by the lower baseline fluorescence and the higher resistance to chemical denaturation in the modified conformational stability assay (Fig. 9). This biophysical property could impair degradation of slow kinetic fibrils and affect cellular seeding; however, this is only hypothetical and has to be evaluated in future studies.

Adequate models for human neurodegenerative diseases are the prerequisite to develop successful therapies. Common approaches include synthetic aggregates of the respective proteins. In PD, synthetic pre-formed fibrils (PFF) of aSyn are well established, sometimes in combination with aSyn overexpression. In extension to these approaches, the fibrils obtained by rSAA can provide virtually limitless amounts of standardized aggregates that are conceivably closer to the human disease pathology and likely more homogenous than PFF (Fig. 10).

### Limitations

This study has several limitations: The extent and quality of clinical data is heterogeneous and we only had a small number of follow-up CSF samples available. Therefore, validation in large and well-characterized cohorts is necessary. Data analysis and sample collection were performed only at a single site, and findings need to be reproduced by further laboratories. Furthermore, cryogenic electron microscopy may elucidate the structural differences between the fibril types. Yet, our findings only allow statements about the structure of amplification products. The structure of the original seed in patient CSF can only be inferred indirectly.

## Conclusions

Taken together, our work provides evidence that the clinical heterogeneity in patients with PD may result from different strains of aSyn pathology. These strains can be detected in living patients by rSAA from CSF. Detecting aSyn strains could potentially be relevant for prognosis and the response to novel therapies directed against aSyn pathology. In addition, using patient-derived aSyn fibrils from the rSAA could improve disease models. Finally, this approach may be applicable to other neurodegenerative diseases, such as Alzheimer´s disease, where the potential relevance of different strains is already implicated by the analysis of post mortem brain samples [39].

## Electronic supplementary material

Below is the link to the electronic supplementary material.

Supplementary Material 1

## Data Availability

No datasets were generated or analysed during the current study.

## Data and materials

Correspondence and requests for materials should be addressed to BHF.
