# Supplementary material for: Recursive seed amplification detects distinct α-synuclein strains in cerebrospinal fluid of patients with Parkinson’s disease
Source: Acta Neuropathol Commun. 2025 Jan 20;13:13. doi: 10.1186/s40478-024-01923-8 (PMC11749544; doi:10.1186/s40478-024-01923-8)
Supplement: Supplementary file 1 — Supplementary Material 1 [file 40478_2024_1923_MOESM1_ESM.pdf]

Supplementary Figures and Tables for

Recursive seed amplification detects distinct α-synuclein strains in cerebrospinal fluid of patients with Parkinson's disease

Stefan Bräuer, Iñaki Schniewind, Elisabeth Dinter, Björn H. Falkenburger*

*Corresponding author: bfalken@ukdd.de

This file includes:

Supplementary Figures 1 to 6
Supplementary Tables 1 to 2

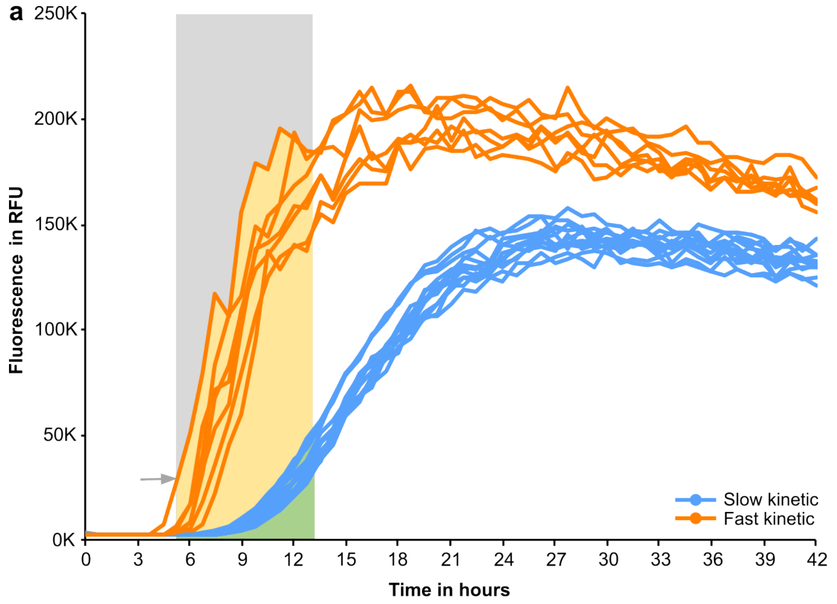

	Timepoint first sample reaches RFU value >10,000	5.25 h
	Interval for AUC calculation	5.25 h - 13.25 h (8h)
	Max. AUC	2,931,000,000
	Threshold AUC	1,172,400,000
	Min. AUC fast kinetic samples	1,581,000,000
	Max. AUC slow kinetic samples	382,909,000

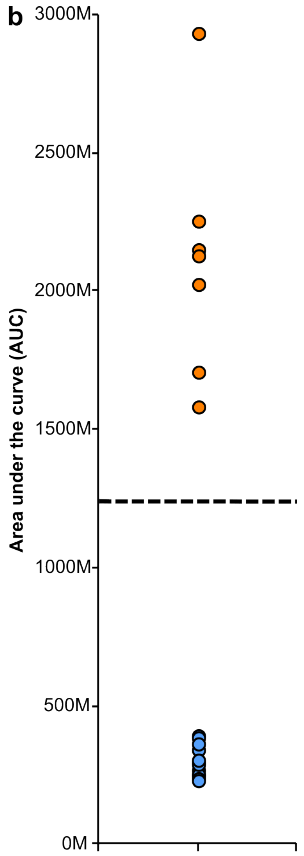

Supplementary Fig. 1. Definition of the fast and slow kinetic types. a The curves represent the average of four technical replicates. An eight-hour interval (indicated as grey area) was defined, starting with the first time the fastest sample reached a fluorescence value higher than 10,000 RFU (grey arrow). For each fibril, the average area under the curve (AUC) in this interval was calculated. For each experiment, a threshold was defined as 40% of the maximum AUC (yellow area) in that experiment. Fibrils with a greater AUC were termed fast kinetic (orange), fibrils with a lower AUC were termed slow kinetic (blue). The threshold of 40% was determined empirically; it generally showed good distinction between the kinetic types. **b** Every dot represents the average AUC in the 8h interval for the curves depicted in (a). The 40% threshold is depicted as a black dashed line.

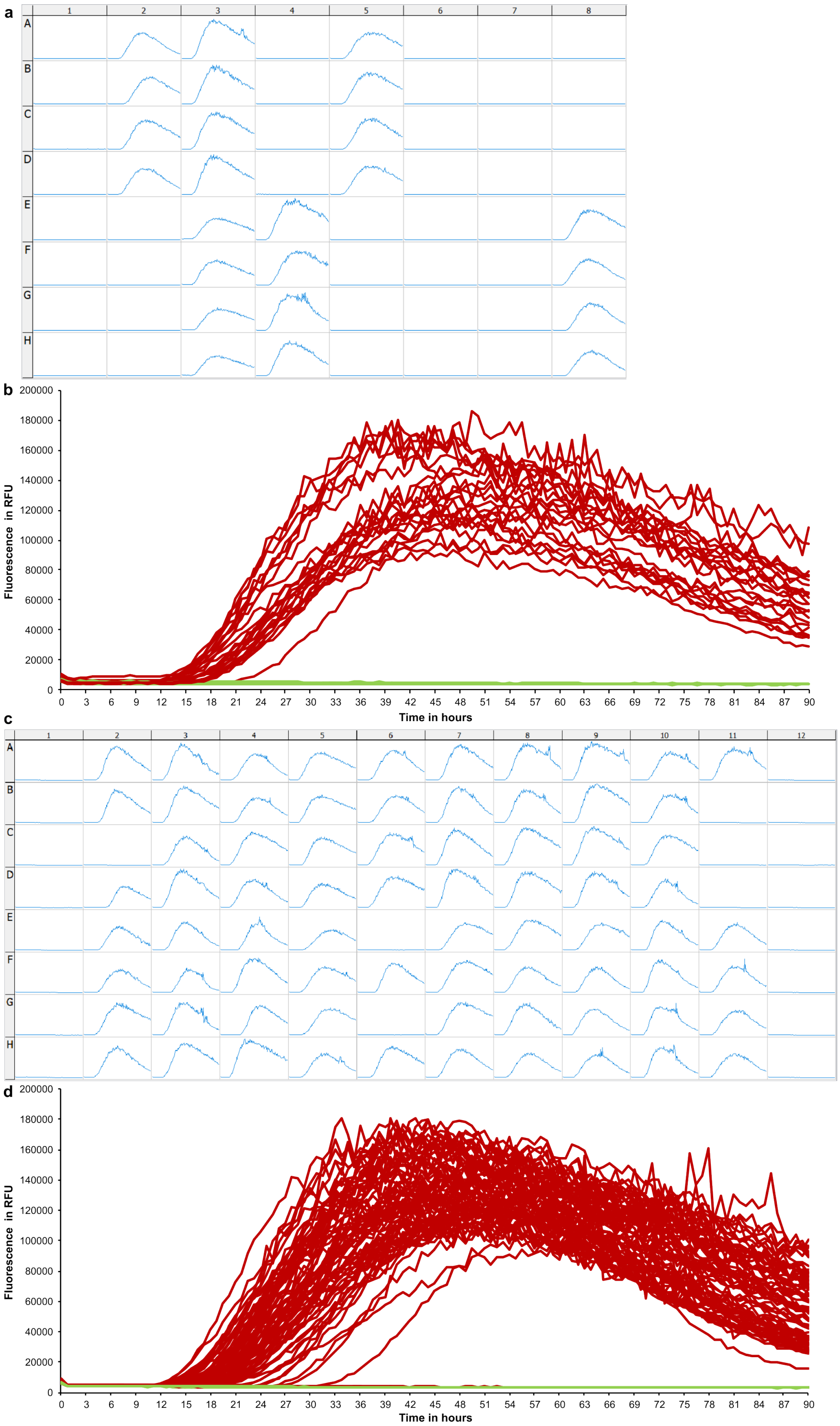

Supplementary Fig. 2. Synuclein batch quality control for strain detection. a Example of a batch validation. Every sample is run in quadruplicates (xA-xD; xE-xH). All positive controls (wells 2A-D, 3A-D, 3E-H, 4E-H, 5A-D, 8E-H) show aggregation, no negative controls (wells 1A-D, 1E-H, 2E-H, 4A-D, 5E-H, 6A-D, 6E-H, 7A-D, 7E-H, 8A-D) show any increase in fluorescence. **b** Curves from (a), positive controls are coded in dark red, negative controls in light green. No aggregation is observed in any negative control in a 90h interval. All replicates of all positive controls reach the threshold in 30h interval. **c** Example of an amplification experiment (Amp1) from the CSF of patients with PD. As in (a), samples are run in quadruplicates (wells xA-xD; xE-xH). **d** Curves from (c), PD samples are coded in dark red, negative controls in light green. No aggregation is observed in any negative control in a 90h interval.

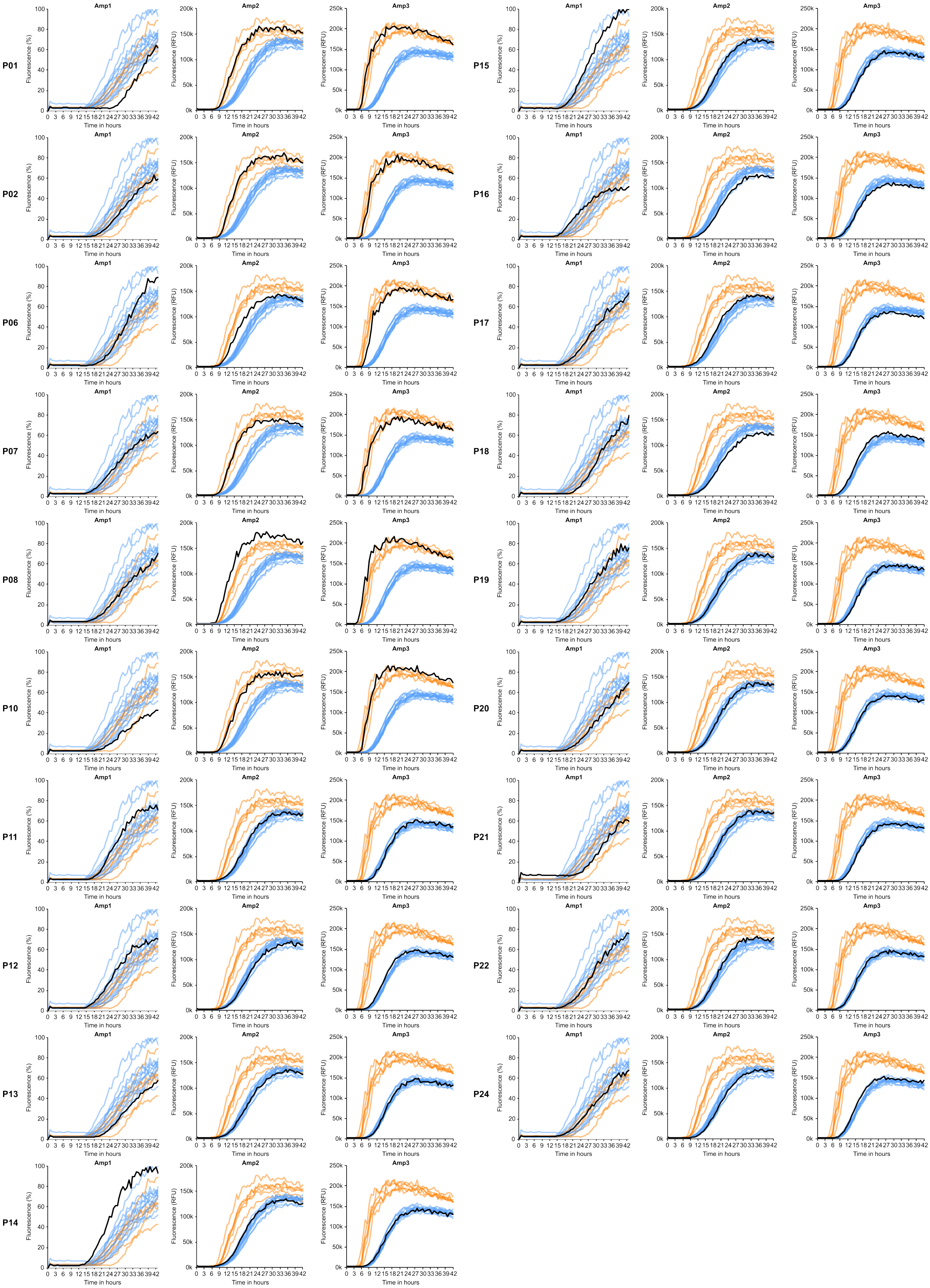

Supplementary Fig. 3. Exemplary rSAA kinetics of individual patients. In each line, this figure highlights the aggregation curves of one patient in black. See also Fig. 1

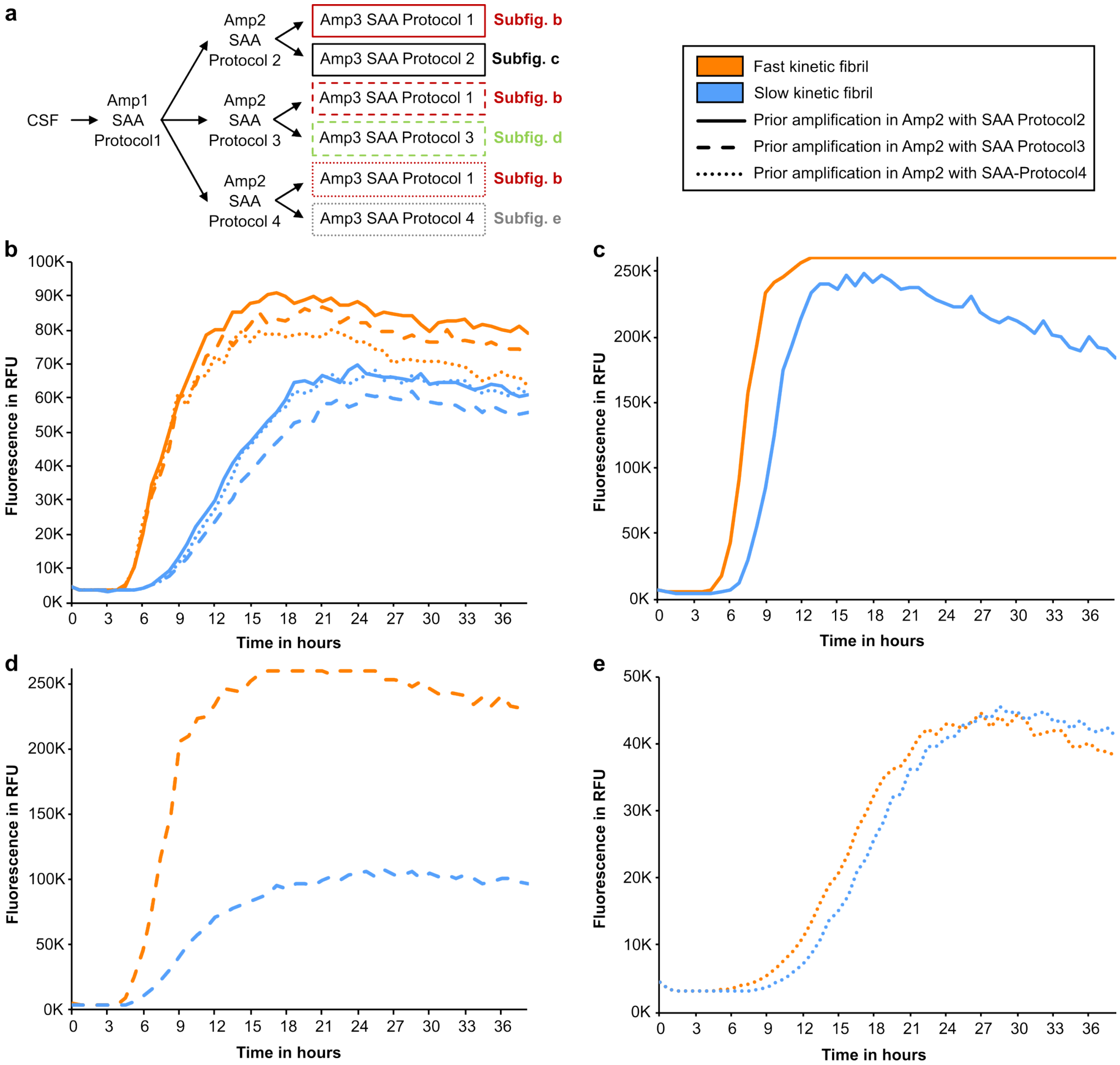

Supplementary Fig. 4. Kinetic differences are preserved in standard SAA even after interposed differing assay protocols. a Schematic overview of the experiments. In addition, fibrils from Amp1 were separately amplified as described in Figure 1 to test their kinetic type. Second round of amplification (Amp2) was performed with different assay protocols (SAA Protocol 1: standard protocol as described in the method section; SAA Protocol 2: no SDS, 100mM PIPES pH 6.0 instead of 40mM PB pH 8.0, 350mM NaCl instead of 170mM; SAA Protocol 3: without SDS, 350mM NaS2O3 instead of 170mM NaCl; SAA Protocol 4: without SDS). The Amp2 products were amplified in a third round (Amp3) using the standard SAA-protocol (b) and, only for exemplary illustration, the same protocol as for Amp2 (c-e). **b** Amp3 results using the standard SAA Protocol 1 with the Amp2 products from the three additional protocols (SAA Protocols 2-4) as seeds. The kinetic type is unchanged after interposed differing SAA protocols. Panels c-e are only exemplary, to illustrate how the kinetic could look in other assay protocols. **c** Amp3 results using the additional SAA Protocol 2 with the Amp2 products from SAA Protocol 2 as seeds. **d** Amp3 results using the additional SAA Protocol 3 with the Amp2 products from SAA Protocol 3 as seeds. e. Amp3 results using the additional SAA Protocol 4 with the Amp2 products from SAA Protocol 4 as seeds. In (b-e) orange lines represent fast kinetic fibrils (P19), blue lines represent slow kinetic fibrils (P5). Solid lines represent fibrils amplified with SAA Protocol 2 in Amp2, dashed lines represent fibrils amplified with SAA Protocol 3 in Amp2, and dotted lines represent fibrils amplified with SAA Protocol 4 in Amp2.

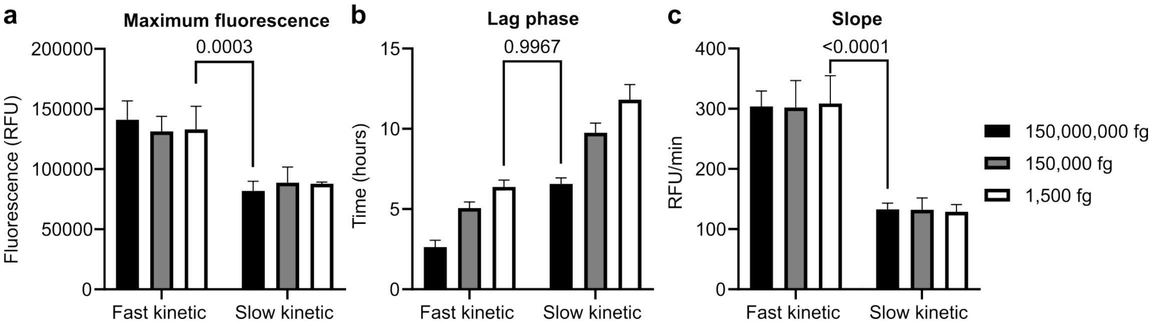

Supplementary Fig. 5. Influence of differing fibril concentrations on kinetic parameters. Kinetic parameters of aggregation curves depicted in Fig. 6. Data are mean ± standard deviation. P values from two-way ANOVA with Tukey's correction for multiple comparisons.

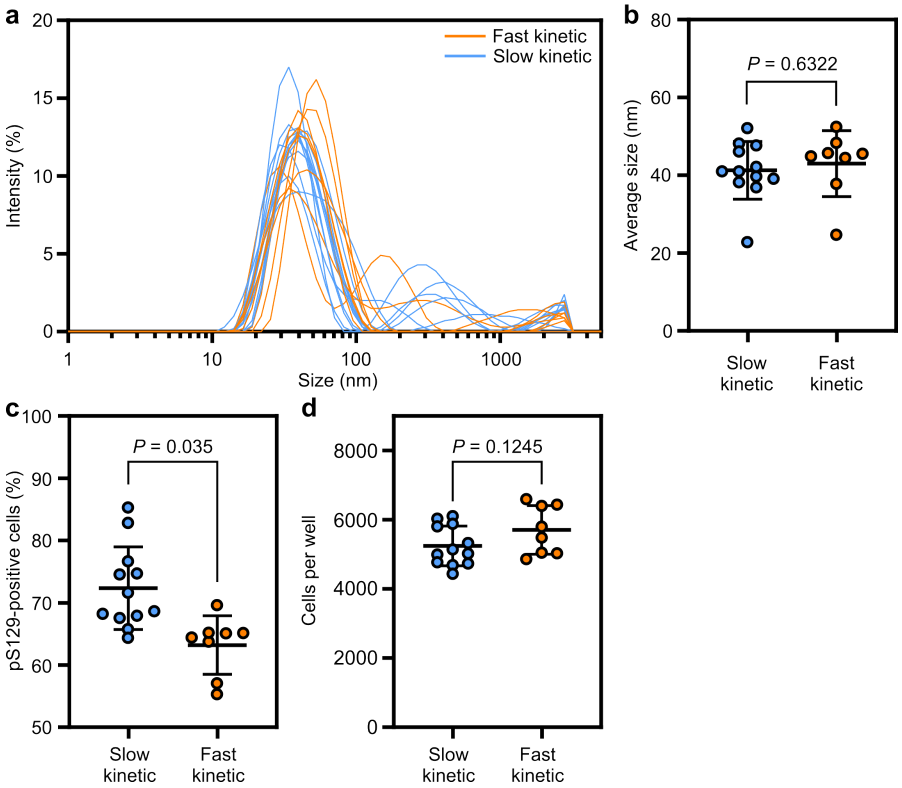

Supplementary Fig. 6. Biophysical and biological properties of patient-derived aSyn fibrils. a Representative dynamic light scattering measurements of patient-derived fibrils after sonication. **b-e** Comparison of fibrils with fast (n = 8) versus slow (n = 12) recursive seed amplification assay (rSAA) kinetics, regarding average size after sonication (b), proportion of cells containing pS129-positive particles (c), and number of imaged and analyzed cells per well (d). Each dot represents one patient. Lines represent mean ± standard deviation, *P* value from two-tailed unpaired *t*-test.

Demographics	Fast kinetic	Slow kinetic
Age at onset in years	56.1 (5.9)	54.9 (13.0)
Age at LP in years	60.7 (9.0)	60.0 (12.6)
Duration at LP in years	4.5 (3.7)	4.1 (2.9)
MoCA at LP	26.4 (3.9)	25.5 (3.5)
UPDRS III at LP	19.9 (12.2)	19.7 (11.2)
LEDD at LP	435.6 (439.6)	471.7 (376.5)
UPDRS III at disease duration 3-5 years	16.2 (4.6)	19.0 (6.4)
LEDD at disease duration 3-5 years	375.0 (373.8)	556.1 (410.5)
Gender (female/male)	2/6	6/6

Supplementary Table 1. Demographics (entire cohort). Parameters: Fast kinetic female, fast kinetic male, slow kinetic female, slow kinetic male. LEDD: Levodopa daily dose, LP: Lumbar puncture, MoCA: Montreal cognitive assessment, UPDRS III: Unified Parkinson’s disease rating scale part III (motor). Data depicted as mean (standard deviation).

Patient	No. of clones	No. of experiments per clone	Total no. of images	Total no. of analyzed cells
P01	3	4	288	197,204
P02	3	7	504	318,063
P03	3	5	360	309,054
P04	3	6	432	356,445
P06	3	5	360	308,056
P07	3	6	432	333,836
P08	3	7	504	281,284
P10	3	4	288	222,409
P11	3	4	288	185,325
P12	3	4	288	236,172
P13	3	5	360	227,265
P14	3	4	288	174,310
P15	3	8	576	416,119
P16	3	4	288	242,363
P17	3	4	288	225,518
P18	3	4	288	159,735
P19	3	4	288	235,556
P20	3	3	216	154,044
P21	3	4	288	188,591
P22	3	4	288	203,087

Supplementary Table 2. Automated analysis of cell experiments. Each sample of patient-derived fibrils was transfected into three separate and independently cultured HEK cell clones overexpressing aSyn and GFP in 3-8 independent experiments, resulting in the acquisition of a total of 6,912 images and the analysis of nearly 5 million cells throughout all experiments.
